# A Modular Health-Related Quality of Life Instrument for Electronic Assessment and Treatment Monitoring: Web-Based Development and Psychometric Validation of Core Thrive Items

**DOI:** 10.2196/12075

**Published:** 2019-01-25

**Authors:** Paul Wicks, Stacey McCaffrey, Kim Goodwin, Ryan Black, Michael Hoole, James Heywood

**Affiliations:** 1 PatientsLikeMe Cambridge, MA United States

**Keywords:** personal health records, health-related quality of life, patient reported outcome measures

## Abstract

**Background:**

Patient-reported outcome (PRO) measures describe natural history, manage disease, and measure the effects of interventions in trials. Patients themselves increasingly use Web-based PRO tools to track their progress, share their data, and even self-experiment. However, existing PROs have limitations such as being: designed for paper (not screens), long and burdensome, negatively framed, under onerous licensing restrictions, either too generic or too specific.

**Objective:**

This study aimed to develop and validate the core items of a modular, patient-centric, PRO system (*Thrive*) that could measure health status across a range of chronic conditions with minimal burden.

**Methods:**

Thrive was developed in 4 phases, largely consistent with Food and Drug Administration guidance regarding PRO development. First, preliminary core items (common across multiple conditions: *core Thrive items*) were developed through literature review, analysis of approximately 20 existing PROs on PatientsLikeMe, and feedback from psychometric and content experts. Second, 2 rounds of cognitive interviews were iteratively conducted with patients (N=14) to obtain feedback on the preliminary items. Third, core Thrive items were administered electronically along with comparator measures, including 20-item Short-Form General Health Survey (SF)-20 and Patient Health Questionnaire (PHQ)-9, to a large sample (N=2002) of adults with chronic diseases through the PatientsLikeMe platform. On the basis of theoretical and empirical rationale, items were revised or removed. Fourth, the revised core Thrive items were administered to another sample of patients (N=704) with generic and condition-specific comparator measures. A psychometric evaluation, which included both modern and classical test theory approaches, was conducted on these items, and several more items were removed.

**Results:**

Cognitive interviews helped to remove confusing or redundant items. Empirical testing of subscales revealed good internal consistency (Cronbach alpha=.712-.879), test-retest reliability (absolute intraclass correlations=.749-.912), and convergent validity with legacy PRO scales (eg, Pearson r=.5-.75 between Thrive subscales and PHQ-9 total). The finalized instrument consists of a 19-item core including 5 multi-item subscales: *Core symptoms*, *Abilities*, *Mobility*, *Sleep*, and *Thriving*. Results provide evidence of construct (content, convergent) validity, high levels of test-retest and internal consistency reliability, and the ability to detect change over time. The items did not exhibit bias based on gender or age, and the items generally functioned similarly across conditions. These results support the use of Thrive Core items across diverse chronic patient populations.

**Conclusions:**

Thrive appears to be a useful approach for capturing important domains for patients with chronic conditions. This *core* set serves as a foundation to begin developing modular condition-specific versions in the near future. Cross-walking against traditional PROs from the PatientsLikeMe platform is underway, in addition to clinical validation and comparison with biomarkers. Thrive is licensed under Creative Commons Attribution ShareAlike 4.0.

## Introduction

### Patient-Reported Outcomes

Patient-reported outcomes (PROs) are reports of health status that come directly from the patient and are typically captured via a questionnaire that has been developed with clearly defined methods, provides proof of validation, and has instructions for use [1]. PROs are one method of incorporating patient perspectives into drug development [2], such as helping to identify trade-offs between treatment characteristics and health-related quality of life (HRQoL) [3]. Accordingly, academic researchers, clinicians, pharmaceutical manufacturers, and their contract research organizations have developed over a thousand PROs over the past few decades with the intent to use some of them as endpoints within clinical trials [4,5]. PROs include single-domain and multi-domain instruments covering a diverse array of domains including overall health status, condition impact, HRQoL, mood, pain, functioning, medication adherence, and treatment side effects.

In addition to their use in trials, a subset of (mostly specialist) clinics deploy PROs during routine clinical practice to help monitor patient symptoms and functioning and to assist with decision making. The incorporation of PROs into electronic medical records is likely to accelerate this trend [6]; their use for symptom management has been particularly successful in oncology [7]. Routine use of remote symptom monitoring is associated with clinically significant benefits in HRQoL, fewer admissions, and even overall survival, probably via improved communication with health care professionals [8].

Whereas other medical tools such as continuous glucose monitors were once the preserve of specialist clinics to check on patient compliance, today people with diabetes themselves are using these tools and integrating them into self-coded apps and jury-rigged mechanisms to develop their own *closed-loop open artificial pancreas* [9]. It should be no surprise, then, that some patients and caregivers harness PROs, research tools originally designed to monitor the outcomes of whole groups of patients in clinical trials, and use them to understand their own individual progress with disease, put themselves into context, self-experiment, and even conduct citizen-science experiments [10]. With the right support, some patients have even developed their own PROs to deal with the frustrations they have encountered with repurposing tools to suit their needs [11].

That was part of the motivation behind the development of the online community PatientsLikeMe, which was first founded in 2005. One feature of the site allows people living with amyotrophic lateral sclerosis (ALS) to access a patient-reported version of the clinician-reported outcome (ClinRO) used in clinical research to characterize patient function, the ALS functional rating scale revised (ALSFRS-R [12]). At the time PatientsLikeMe was launched, ALS researchers were advised not to tell research participants their own ALSFRS-R scores or how they were doing relative to other patients like them [13]. Patients tracking their own ALSFRS-R scores on the site could see their progression overlaid on percentile curves of other patients like them (with different curves for slower ALS subtypes such as progressive muscular atrophy and primary lateral sclerosis) and bring these data to clinic appointments with their health care professionals, helping to improve communication and management [14]. At first, there was concern that PROs might lack resolution and accuracy relative to ClinROs, yet subsequent validation studies have found a high degree of agreement (eg, Spearman rho=.965, *P≤*.001 [15]).

### Limitations of Patient-Reported Outcomes for Digital Health Apps

However, as PatientsLikeMe expanded to other conditions such as multiple sclerosis (MS), Parkinson disease (PD), HIV, mood disorders, fibromyalgia, epilepsy, autism spectrum disorder, and organ transplants, it became clear that the state of PRO development was highly uneven across these conditions. While some PROs focused on symptoms and pathological elements of disease, others focused on the impact of the condition, treatment side effects, or broader concepts such as HRQoL. As standards on the quality of PRO development (such as the Food and Drug Administration’s (FDA’s) guidance for industry on PRO development in labeling [16]) became available, it also became clear that the psychometric quality and rigor of instruments varied enormously, with some meeting only low standards of reliability, having had little input from patients themselves, or undergoing little in the way of psychometric validation for responsiveness to change, clinically important differences, or conformity to modern psychometric methods such as Rasch modeling [17]. In addition to well-worn limitations identified in the psychometric field [17], we identified a range of issues that may not have arisen in traditional clinical settings but are problematic for their use in digital health apps for both patients (Table 1) and professionals (Table 2).

### Objectives of This Study

Adapting what we felt were the best approaches from the PRO field, we sought to develop a *modular* questionnaire system that addressed the limitations we had identified for their use in real-world and digital health apps. Specifically, we aimed to develop a set of questions that covered the key domains of HRQoL in adults with chronic illness that was brief, minimally burdensome, positively framed, and that could interleave additional items to account for comorbidity in future condition-specific modules.

Methodologically, we sought to conform (to the extent possible) with the FDA’s Guidance for Industry for PRO development [16] by completing the following objectives:

1. Developing a conceptual framework and the preliminary item pool through literature review and expert input
2. Cognitive debriefing of draft items with participants
3. Revising these items and framework accordingly
4. Collecting data and evaluating psychometric properties (such as rating scale functioning, reliability, convergent validity, ability to detect change, and bias)
5. Modifying the instrument based on results of the empirical evaluation
6. Collecting data and analyzing psychometric properties of the revised instrument
7. Finalizing the instrument and scoring

**Table 1:** Issues identified by the team for patients with the patient-reported outcome status quo.

Issues for patients	Example in existing PROs^a^	Implications	Proposed solution	Implementation in Thrive
PROs ignore comorbidity	For example, SF-36^b^ does not contain important domains for a specific chronic condition, whereas condition-specific instruments are unclear on how user should dissociate primary condition from comorbidities	Typical PatientsLikeMe user has a median of 3 moderate-serious medical conditions; fielding additional PROs for each condition dramatically increases burden and redundancy	Core Thrive items asked of all users; curated set of additional symptoms, abilities, and thriving items fielded according to reported conditions	Core Thrive item asks separately about impact of each condition and comorbidity independently, for example, “Parkinson’s impact=a lot” but “Eczema=not at all”
No personalization for the individual	Redundant questions, for example, pregnancy in males. At best, there are instructions to skip irrelevant questions (eg, “If no, skip to 12”)	Patients wade through the same clumsy skip logic instructions (or irrelevant questions) over and over again	Let patients specify once that something is not relevant and remember that in the future	Option of “Stop asking me this” checks why patient wants to skip and asks if we can assume the last answer given will continue being the same
Large number of questions	For example, autism treatment evaluation checklist contains 78 items	Takes a long time to complete (approximately 10 seconds per item) and may cause drop-off	Ask as few questions as possible	Review of literature and patient-submitted data to identify most common issues
Long question stems and responses	Parkinson disease rating scale requires reading 1456 words	Difficult to read on mobile screens, may require scrolling, risks biasing answers	Use brief, active voice items and consistent response scales rather than longer text-anchored responses	Items are Likert-style unipolar responses
Negative framing	For example, Beck Depression Inventory: “(0) I don't feel disappointed in myself (1) I am disappointed in myself (2) I am disgusted with myself (3) I hate myself”	Fails to identify, for example, users who feel good about themselves; ignores islands of resilience and important self-expression for users; not appealing to use repeatedly	Frame items in a positive or at least neutral way when possible	Abilities stem asks, “how well could you” and Thriving stem asks, “how often could you”
Variable or unclear recall periods	Recall periods may be missing, “past week” vs “past 7 days”, or very long, for example, past 12 months or “since you were diagnosed”	Different user needs require different recall periods	Codify and test different response periods flexibly, that is, “In the past <recall period> how well could you <activity>?”	Initial validation study developed with “last month” recall period but future work will test other recall periods
Potentially sexist items	For example, fibromyalgia impact questionnaire focuses on disease preventing patient from doing shopping, laundry, and housework	Risks offending users. Also ignores modern options such as home grocery delivery	Avoid making assumptions about how people live their lives with or without illness	Provide general role function items, for example, “responsibilities” or “personal needs” rather than specific chores
Anachronistic items	For example, adolescent systemizing spectrum quotient asks about “programming a video recorder”	Unclear how users will interpret such items; potential for user frustration	Focus on personally defined impact of condition rather than task completion	Use *evergreen* items such as walking or sleeping
Confusing scores and directionality across conditions PROs	For example, scores such as the ALSFRS-R have an arbitrary range 0-48, Unified Parkinson’s Disease Rating Scale is 0-199; sometimes higher is worse, sometimes lower	Difficult for patients to understand meaning; conveys false sense of an interval or ratio level scale	Use a score based on a more relatable frame of reference, for example, 0-10	10-point scales are more familiar

^a^PRO: patient-reported outcome.

^b^SF-36: short-form 36 questionnaire.

**Table 2:** Issues identified by the team for professionals with the patient-reported outcome status quo.

Issues for professionals	Example in existing PROs^a^	Implications	Proposed solution	Implementation in Thrive
Incomplete documentation	Most instruments lack detailed instructions for missing data	Unclear how to score, where more validation work is needed, whether items contain bias	Digitize and share item-level response characteristics through data repositories	Work in progress
Onerous licensing restrictions	For example, license-holders of *Morisky medication adherence scale* have threatened lawsuits, demanded fees, and required retractions for an 8-item questionnaire	Risk of litigation restricts innovation. Digital health practitioners may need to adapt licensed instruments to their own needs without wanting to revalidate entire instrument.	All PROs should be licensed under *Creative Commons ShareAlike* to promote scientific dissemination and innovation so that anyone can use and modify them, for free, forever	All Thrive items and supporting documentation are licensed under Creative Commons ShareAlike 4.0

^a^PRO: patient-reported outcome.

## Methods

**Figure 1:**
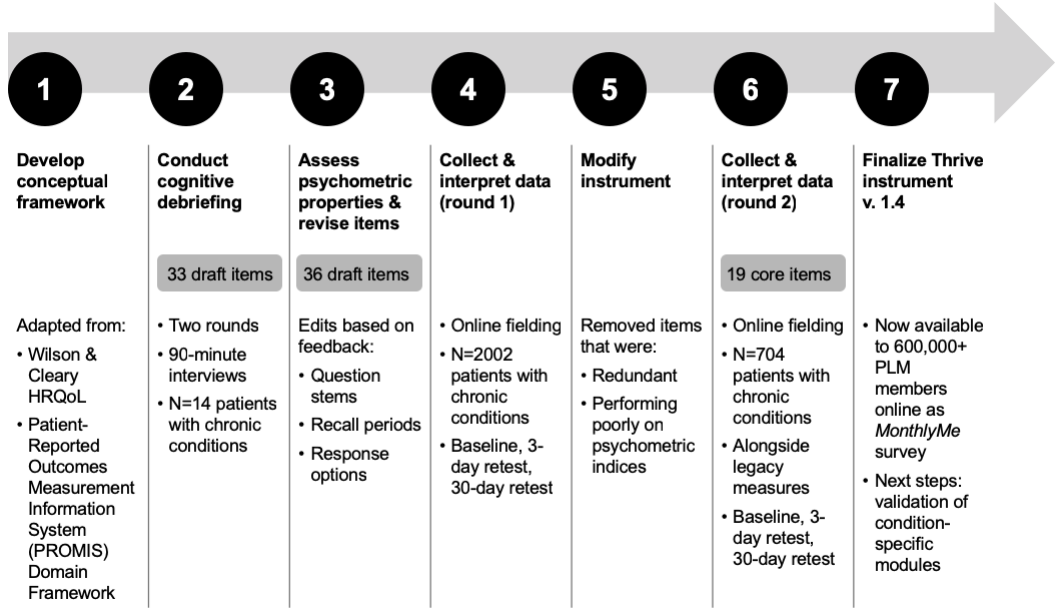
Overview of validation process, adapted from the Food and Drug Administration (2009) guidance for industry. PLM: PatientsLikeMe.

Each phase of the instrument development and validation study is presented in temporal sequence below (Figure 1).

### Setting

Participants were recruited from the membership of PatientsLikeMe.com, an online community for patients living with chronic illness. Potential members are made aware of the site through a variety of channels including Web-based advertising, nonprofit partners, word of mouth, and search. Members join the site with a goal to find other patients like them, track their condition over time, and to benefit from the shared experiences of other members like them [18]. The site is currently only available in English, with most participants living in the United States. Participants were not offered any reimbursement for participating in this study. As a convenience sample of chronic online patients, this group is representative of digital health patients, but caution should be taken in generalizing these findings to other groups.

### Ethical Approval

On request for ethical independent review board, this research was exempted from further ethical review by the New England Independent Review Board as a minimal risk study (WO 1-2559-1).

### Developing a Conceptual Framework

A literature search was conducted to guide the development of a preliminary conceptual model and item generation. Consistent with widely regarded conceptual models [19,20], HRQoL was considered to be a broad and dynamic construct that incorporates quality of life, general health perceptions, functional status, symptoms, as well as intraindividual and environmental factors.

Each of these aspects was considered when developing the initial item pool to ensure that the final Thrive core items adequately captured HRQoL. In particular, we were influenced by the Patient-Reported Outcomes Measurement Information System (PROMIS) Domain Framework [21] and prospectively sought to develop items relevant to physical health (including common symptoms such as pain, fatigue, and sleep disturbance), mental health (including mood symptoms, cognitive dysfunction, and positive psychology), and social health (including ability to participate and social isolation). The research team, which included content experts and psychometricians, collaboratively drafted the preliminary Thrive items, some of which were adapted from validated instruments and published PRO HRQoL tools (eg, the *SF-1* general health item from the Research and Development (RAND) Corporation SF-36 [22]).

### Item Development

A PRO instrument consists of instructions, items (which incorporate a recall period), and the items’ response options. Given the focus on chronic health conditions, we settled on a *last month* response window. Although a *30-day* response window may appear more precise, we aimed for questions to seem conversational. Since we planned to code questions as medical objects in a database to support use across multiple platforms, wherever possible, we tried to take a consistent approach to question stems and response options.

*Symptoms* were defined as any physical or mental feature regarded as indicating a condition or disease, particularly when such a feature was apparent or bothersome to the patient. On the basis of the World Health Organization’s International Classification of Function [23], we offered participants a consistent symptom question type: “Please rate the severity of any <SYMPTOM ITEM> over the past month” and response options: *None*, *Mild*, *Moderate*, or *Severe*.

*Abilities* were defined as the degree to which a participant possessed the means to do something important to them, particularly to function independently. On the basis of our aim to offer positively framed question stems, we phrased these as “Over the last month, how well could you <ABILITY ITEM>?” with response options of *Extremely well*, *Very well*, *Fairly well*, *Poorly*, or *Not at all*. In this way, we aimed to identify participants who were functioning particularly well on some items despite their condition as well as to make the experience of taking the instrument a more pleasant one, and to avoid floor or ceiling effects.

*Thriving* was defined as the extent to which a participant was living the life they wanted to lead, regardless of their health status. These were phrased “Over the last month, how often did you <THRIVING ITEM>?” with a response scale of *All of the time*, *Most of the time*, *Some of the time*, or *None of the time*.

### Cognitive Interviews

#### Procedures

Cognitive interviews were conducted to gather qualitative feedback regarding the preliminary items and to establish content validity. A total of 2 interviewers trained in cognitive interviewing procedures completed the interviews individually with participants over the phone. Interviews were not audio-recorded and lasted approximately 90 min. Retrospective probing was used to enhance realism [24], and interviewers followed a semistructured interviewing script that allowed for deviation as appropriate. Cognitive interviews were conducted in 2 rounds so that content modified following the first round of interviews could be evaluated in a second round.

#### Participants

As one of the main objectives was to create a system that would replace the legacy PROs on the PatientsLikeMe website, to ensure that the items were reviewed by a diverse patient group living with chronic health conditions who were representative of our most populated communities, members of PatientsLikeMe who met the following study inclusion criteria were invited to participate:

1. Reported a primary condition of ALS, PD, multiple sclerosis (MS), major depressive disorder (MDD), generalized anxiety disorder (GAD), or posttraumatic stress disorder (PTSD)
2. Aged 18 years or older
3. Primarily resided in the United States

### Empirical Evaluation

Following cognitive interviews, the draft core Thrive items were programmed in PatientsLikeMe’s research survey tool (RST) and administered along with validated comparison measures (PHQ-9 and the Medical Outcomes Study SF-20) to patients with chronic medical conditions (*Round 1*). On the basis of the items’ psychometric functioning and expert input, items were revised or removed. The updated Thrive instrument was again administered to an independent sample of patients (*Round 2*) alongside validated generic comparison measures (PHQ-9, SF-20) and PROs offered to patients on the PatientsLikeMe website with at least some psychometric validation (multiple sclerosis rating scale, MSRS) for participants with MS, ALSFRS-R for participants with ALS, and PatientsLikeMe-QoL for participants with systemic lupus erythematosus (SLE). Additional PROs used on PatientsLikeMe were fielded (Parkinson’s disease rating scale [PDRS] in PD and *mood map* in mood disorders) but because of a lack of previous psychometric validation, they are not reported here.

During both rounds, participants were asked to complete assessments at 3 timepoints:

1. (Administration 1) Thrive + comparator measures, baseline
2. (Administration 2) Thrive only: 3 days after Administration 1, for evaluating stability
3. (Administration 3) Thrive + comparator measures: 30 days after Administration 1, for evaluating ability to detect change over time

### Materials-Comparator Patient-Reported Outcomes

#### Patient Health Questionnaire-9: All Participants

The PHQ-9 is a 9-item self-report measure of depression based on the Diagnostic and Statistical Manual, Fourth Edition diagnostic criteria [25]. It has been validated for use with primary care, obstetrics/gynecological patients, and the general population, and has been found to be useful as both a clinical and research tool [25-27]. It has also demonstrated sensitivity to detect change in depression status over time in medical outpatients [28].

#### Short-Form General Health Survey -20: All Participants

SF-20 is a brief self-report health survey that captures 6 health concepts: physical functioning, role functioning, social functioning, health perceptions, pain, and mental health [29]. The SF-20 has exhibited adequate levels of reliability and validity in a general population sample and patient population [29,30].

#### Multiple Sclerosis Rating Scale: Participants With Multiple Sclerosis

Inspired by the Guy’s Neurological Disability Scale [31], the 7-item MSRS was developed by PatientsLikeMe to capture the impact of MS on daily living. This scale has demonstrated convergent validity through correlations with walking scores and physician-derived measures [32].

#### Amyotrophic Lateral Sclerosis Functional Rating Scale Revised: Participants With Amyotrophic Lateral Sclerosis

The ALSFRS-R is one of the most widely used instruments to capture ALS disease progression [12]. The ALSFRS-R is correlated with disease progression and survival [33,34], and research has suggested good internal consistency and reproducibility.

#### PatientsLikeMe-Quality of Life: Participants With Systemic Lupus Erythematosus

The PatientsLikeMe-QoL is intended to capture HRQoL related to physical function, mental distress, and social functioning over the past 30 days. This instrument has exhibited high internal consistency and convergent validity [35].

### Power Analysis

The target N for each patient group at administration 2 (3-day retest) was 100. The sample size of 100 was derived from a power analysis to detect a significant difference between an intraclass correlation coefficient of .80 (within the acceptable level) and 0.69 (below the acceptable), assuming 80% power. Specifically, a sample size of 100 would detect whether the CI of the reliability coefficient includes values below the accepted reliability threshold (Rxx=.70) 80% of the time. Notably, because of difficulties with achieving a sufficient sample size during Round 2, results were not evaluated separately by patient group.

### Participants

Adult (18 years or older) PatientsLikeMe members primarily residing in the United States who reported a primary condition of ALS, MS, PD, MDD, GAD, PTSD, or SLE were sent an invitation to participate through the PatientsLikeMe platform. The following information is reported in accordance with the Checklist for Reporting Results of Internet E-Surveys (CHERRIES) checklist [36]. All surveys were voluntary and would not affect invitees’ use of other features on the PatientsLikeMe site. Individual users had a password-protected log-in and could only take the survey once; we have tools to prevent multiple accounts from originating in the same location, including account registration, cookies, and internet provider tracing. No incentives were offered, question order was not randomized, certain items only appeared based on responses to previous questions (ie, were *branching*) to minimize burden, and the total number of questions varied per respondent. There was 1 question per page with a *back* button allowing patients to navigate back 1 page to review their previous response.

On March 23, 2017, 20,941 PatientsLikeMe members fitting the inclusion criteria mentioned above were invited to the Round 1, baseline survey; this survey remained open until April 10, 2017. Participants who did not complete this survey were sent 1 reminder message 3 days after the invitation. Those who completed the survey were automatically sent an invitation to administration 2 three days after completion of administration 1. Administration 2 was open for the same time period as the baseline survey. Those who completed the Round 1 baseline survey were invited to a 30-day retest (administration 3) on May 2, 2017, which remained open until May 10, 2017.

For the second round of the surveys, 12,460 participants were sent invitations on June 15, 2017, to the Round 2 baseline survey, which remained open until July 6, 2017. Reminders and the 3-day test/retest invitation were sent in a manner identical to that of Round 1; Round 2-administration 2 was also open from June 15, 2017, to July 6, 2017. Those who completed the Round 2 baseline were invited to a 30-day retest on July 25, 2017 which remained open until August 10, 2017. All numbers pertaining to Round 1 and Round 2 are reported in the results section.

### Analytic Plan

Psychometric validation is an iterative process that is driven by both theoretical and empirical support; therefore, the Thrive research team provided input and feedback during each step of the validation process. Thrive was evaluated using both classical and modern test theory approaches, including evaluation of: rating scale functioning, dimensionality, person-to-item targeting, bias (gender [male, female], race [white, nonwhite], condition [neurodegenerative, autoimmune relapsing, psychiatric]), internal consistency reliability, test-retest reliability, convergent validity, and ability to detect change using longitudinal data. The primary purpose of the first round of testing was to explore item functioning and to make revisions as necessary before the second round. Analytic procedures for this second round of testing were largely consistent with those utilized in Round 1. Readers are referred to Bond and Fox [37] and Furr [38] for more information about these analyses. Analyses were conducted in SPSS version 24 (IBM Corporation, New York) and WINSTEPS 3.74.0 (Beaverton, Oregon) by author SM.

## Results

### Cognitive Interviews

Twelve participants completed the first round of cognitive interviews. Participants (75% [9/12] female) reported primary diagnoses of MS (33% [4/12]), fibromyalgia (17% [2/12]), GAD (8% [1/12]), MDD (8% [1/12]), ALS (8% [1/12]), bipolar disorder (8% [1/12]), and SLE (8% [1/12]). As cognitive interviews were being conducted, the interviewers regularly met together and with the research team to discuss participant feedback with the goal of identifying recurring themes. Participants identified several items that had redundant content, were too vague and caused confusion, or that they felt were not important for purposes of monitoring their health. Several items were removed or revised based on participants’ suggested rewordings to increase clarity, response options were modified to enhance consistency or reduce confusion, and the recall period was made consistent across items. For example, when probed about a coping question (“How well could you cope over the last month?”), participants expressed confusion (eg, “Cope with what?”) and felt that one’s ability to cope and deal with life stressors was already covered by other items. Similarly, response options of several items were modified for consistency and to reduce confusion. For example, the question wording “How well could you see yourself as a worthwhile person over the last month?” was changed to “Over the last month, how often did you see yourself as a worthwhile person?”

A few respondents wanted to express more detail about pain or sleep, which were issues of particular concern for them. As this core instrument is meant to be applicable to all PatientsLikeMe members, the research team decided to revisit further detail on those issues as future modular additions to the instrument.

A second round of cognitive interviewing was conducted to evaluate the revised content. A total of 2 participants (1 male) completed the second round of cognitive interviews. These participants reported primary diagnoses of bipolar disorder and SLE. Participants provided relatively similar and positive feedback about the items. This feedback was communicated back to the research team and minor revisions to the survey were made.

### Psychometric Evaluation: Round 1

#### Participants

Consolidated Standards of Reporting Trials (CONSORT) flow diagrams are presented in Multimedia Appendix 1. Of the 20,941 PatientsLikeMe members who were sent an invitation to participate, 2311 responded to the invitation by clicking on the survey link, and 86.6% of these members completed administration 1 (N=2002). It took participants approximately 16 min to complete the battery. The survey was open for 17 days in total. To evaluate test-retest reliability, the same 2002 participants were invited to complete the draft core Thrive items 3 days after the first administration. The retest was completed by 924 participants. Finally, the original 2002 participants were invited to complete the battery (Thrive, PHQ-9, SF-20) approximately 30 days after the first and initial administration to evaluate core Thrive items’ ability to detect change over time. In total, 717 participants completed the battery at the 30-day administration.

Demographic and clinical characteristics of this sample are presented in Table 3. The average age of participants was approximately 55 years, and the majority of participants were non-Hispanic, white, and female. The sample was highly educated; 33% of the sample completed some college, 25% completed college, and 19% received postgraduate education.

#### Round 1 Results

The purpose of Round 1 was to explore item functioning and to make revisions as necessary before the second round. A summary of results from Round 1 can be found in Multimedia Appendix 1. Some of the scales evidenced levels of reliability that are below what is typically considered acceptable, some items exhibited bias or poor discrimination. Core Thrive items were modified based on these findings and were subject to empirical evaluation in Round 2.

### Psychometric Evaluation: Round 2

#### Participants

Of the 12,460 participants who were sent an invitation to participate, 887 responded to the invitation by clicking on the survey link, and 79.4% of these participants (N=704) completed the Round 2 baseline survey; 239 completed the 3-day retest and 51 completed 30-day retest. Demographic and clinical characteristics of this sample are presented in Table 4.

#### Round 2 Results

Results are presented by scale below and are summarized in Tables 5-7. The final surviving Thrive items from Round 2 testing are listed in Table 8, and summary of the items that were retained or discarded is provided in Multimedia Appendix 2. Detailed results, including evaluation of dimensionality, item difficulty, fit statistics, response category thresholds, and person-to-item maps, are also presented in Multimedia Appendix 2.

Empirical testing of subscales revealed good internal consistency (Cronbach alpha=.712-.879) and test-retest reliability (absolute intra class correlations=.749-.912). Cronbach alpha for the Sleep subscale was lower (Cronbach alpha=.712), probably owing to the lower count of items.

Convergent validity varied by domain. Correlations were highest between the *Overall Health* Thrive item and *General Health Item* of the SF-20 owing to the similarity of their stem phrasing (Thrive: “Over the last month, how has your health been?”, SF-20: “In general, would you say your health is?”) with the same response options but with different response time periods. The *Impact of Primary Condition* had consistent moderate correlations with all comparator measures (Pearson r=.443-.518). Core symptoms (including anxious mood, depressed mood, fatigue, pain, and stress) had stronger correlations with mental health comparators (Pearson r=.-750-.775 for PHQ-9, SF-20 mental health, PLM-QoL mental subscale) than physical health comparators (Pearson r=.390-.698 for SF-20 physical function, PLM-QoL physical, nonsignificant with ALSFRS-R). The single-item Mobility scale (Walking) had a moderate correlation with physical functioning comparators that themselves contained walking items (SF-20 physical function, MSRS, PLM-QoL physical scale, ALSFRS-R). The Abilities scale correlated most strongly with the PLM-QoL (Pearson r=.770-.809), which asks participants to endorse the extent to which their health limited their ability to participate in physical functioning, mental well-being, or social interaction. Two psychological items (Cognitive and Emotional control) may explain the relatively high correlation with the PHQ-9 (Pearson r=.744). Abilities had a moderate degree of correlation (Pearson r=.450-.520) with comparator measures of physical role function or physical ability (SF-20 physical role, ALSFRS-R). Thriving items were most strongly related to mental health comparators (Pearson r=.743-.806 for PHQ-9, SF-20 mental health, PLM-QoL mental) but had nonsignificant or weak correlations with physical health comparators (Pearson r=.342 for SF-20 physical health, r=.132 *P*=.32 with ALSFRS-R).

Analysis of longitudinal residualized change scores over 30 days found significant, but attenuated, patterns of correlation similar to the results of the convergent validity analysis. The strongest relationship (Pearson r=.496) was between the 2 item-Sleep scale (Falling asleep and Staying asleep) with the single-item PHQ-9 question.

#### Overall Health

Absolute agreement of responses across the 3-day test-retest period (n=239) suggested adequate stability (Table 5). Convergent validity was evaluated by calculating a Pearson correlation between Overall Health and the SF-20 General Health item. Results yielded a strong correlation, providing support for the convergent validity of the Overall Health scale (Table 6). Next, ability to detect change was evaluated by correlating residualized change scores of Overall Health and the SF General Health item over the 30-day testing period. Stated differently, we evaluated the correspondence between change in patients’ responses over time. Results supported the Overall Health scale’s ability to detect change over time (Table 7).

#### Impact of Primary Condition

Absolute agreement of responses to the Impact of Primary Condition item across the 3-day test-retest period was adequate (Table 5). The Impact of Primary Condition scale was related as anticipated to comparator measures, providing support for convergent validity (Table 6). Correlations between residualized change scores (see Table 7) provide support for the Impact of Primary Condition scale’s ability to detect change over time.

**Table 3:** Round 1 participant demographics.

Variable		Baseline	3-day test-retest	30-day retest
Participants (n)		2002	924	717
Age (years), mean (SD)		54.9 (11.6)	56.2 (10.7)	56.0 (11.3)
Conditions, median (range)		2 (1-58)	2 (1-53)	2 (1-58)
**Gender,** **n (%)^a^**				
	Male	600 (30.0)	290 (31.5)	245 (34.2)
	Female	1399 (70.0)	632 (68.5)	471 (65.8)
**Ethnicity,** **n (%)^a^**				
	Hispanic	77 (4.0)	31 (3.5)	26 (3.8)
	Non-Hispanic	1831 (96.0)	861 (96.5)	665 (96.2)
**Race,** **n (%)^a^**				
	Asian	7 (0.4)	1 (0.1)	0 (0.0)
	Black or African American	86 (4.4)	29 (3.2)	23 (3.3)
	Hawaiian	3 (0.2)	2 (0.2)	2 (0.3)
	Native American	25 (1.3)	10 (1.1)	7 (1.0)
	White	1740 (89.6)	821 (91.0)	633 (90.3)
	Mixed	82 (4.2)	39 (4.3)	36 (5.1)
**Education,** **n (%)^a^**				
	8th grade or less	3 (0.2)	0 (0.0)	1 (0.1)
	Some high school	14 (0.8)	8 (0.9)	3 (0.4)
	High school graduate	175 (10.1)	83 (9.6)	66 (9.6)
	Some college	658 (38.1)	305 (35.3)	242 (35.4)
	College	498 (28.9)	254 (29.4)	202 (29.5)
	Postgraduate	378 (21.9)	215 (24.8)	170 (24.9)

^a^Percentage does not include missing cases.

**Table 4:** Round 2 participant demographics.

Variable		Baseline	3-day test retest	30-day retest
Participants (n)		704	239	51
Age (years), mean (SD)		54.5 (11.8)	54.8 (12.1)	53.7 (12.7)
Conditions, median (range)		1 (1-35)	1 (1-27)	1 (1-18)
**Gender, n (%)^a^**				
	Male	189 (26.9)	61 (25.6)	15 (29)
	Female	514 (73.1)	177 (74.4)	36 (70)
**Ethnicity, n (%)^a^**				
	Hispanic	26 (3.9)	7 (3.0)	1 (2)
	Non-Hispanic	640 (96.1)	226 (97.0)	47 (97)
**Race, n (%)^a^**				
	Asian	3 (0.4)	1 (0.4)	1 (2)
	Black or African American	53 (7.8)	13 (5.5)	3 (6)
	Hawaiian	0 (0.0)	0 (0.0)	0 (0)
	Native American	6 (0.9)	2 (0.8)	0 (0)
	White	586 (86.3)	214 (90.7)	39 (81)
	Mixed	31 (4.6)	6 (2.5)	5 (10)
**Education, n (%)^a^**				
	8th grade or less	1 (0.2)	0 (0.0)	0 (0)
	Some high school	6 (1.0)	3 (1.4)	0 (0)
	High school graduate	81 (13.8)	18 (8.6)	7 (17)
	Some college	225 (38.5)	94 (45.0)	15 (37)
	College	160 (27.4)	55 (26.3)	12 (30)
	Postgraduate	112 (19.1)	39 (18.7)	6 (15)

^a^Percentage does not include missing cases**.**

**Table 5:** Reliability estimates for surviving thrive scales.

Thrive scale (number of items)	Internal consistency reliability (Cronbach alpha; n=704)	Test-retest reliability (n=239)	
		Absolute ICC^a^	*P* value
Overall Health (1)	—^b^	.749	<.001
Impact of Primary Condition (1)	—	.763	<.001
Core Symptoms (5)	.815	.909	<.001
Mobility (1)	—	.898	<.001
Sleep (2)	.712	.833	<.001
Abilities (5)	.853	.912	<.001
Thriving (4)	.879	.889	<.001

^a^ICC: intraclass correlation coefficient.

^b^Not applicable.

**Table 6:** Ability to detect change (Pearson correlations between Thrive and comparator instruments’ residualized change scores in longitudinal data, N=51).

Thrive scale item	Pearson r; *P* value										
	PHQ^a^-9 (n=704)	SF^b^-20 (n=704) General Health Item	SF-20 (n=704) Mental Health	SF-20 (n=704) Physical Functioning	SF-20 (n=704) Role Functioning	SF-20 (n=704) Health Perception	MSRS^c^ (n=255)	PLM-QoL^d^ (n=64) Physical	PLM-QoL (n=64) Mental	PLM-QoL (n=64) Social	ALS FRS-R^e^ (n=60)
Overall Health (1 item)	—^f^	.813; <.001	—	—	—	—	—	—	—	—	—
Impact of Primary Condition (1 item)	.463; <.001	—	−.445; <.001	−.439; <.001	−.443; <.001	−.518; <.001	.452; <.001	−.573; <.001	−.492; <.001	−.477; <.001	−.477; <.001
Core Symptoms (5 items)	.750; <.001	—	−.759; <.001	−.390; <.001	−.392; <.001	−.644; <.001	.574; <.001	−.698; <.001	−.775; <.001	−.675; <.001	−.148; .26
Mobility (1 item)	—	—	—	.415; <.001	—	—	−.471; <.001	.687; <.001	—	—	.423; <.001
Sleep (2 items)	−.562; <.001	—	—	—	—	—	—	—	—	—	—
Abilities (5 items)	−.744; <.001		.708; <.001	.478; <.001	.520; <.001	.671; <.001	−.687; <.001	.791; <.001	.770; <.001	.809; <.001	.450; <.001
Thriving (4 items)	−.743; <.001	—	.780; <.001	.342; <.001	.378; <.001	.626; <.001	−.453; <.001	.639; <.001	.806; <.001	.736; <.001	.132; .32

^a^PHQ: Patient Health Questionnaire.

^b^SF: Short-Form General Health Survey.

^c^MSRS: multiple sclerosis rating scale.

^d^QoL: quality of life.

^e^ALSFRS-R: amyotrophic lateral sclerosis functional rating scale-revised.

^f^Not applicable.

**Table 7:** Ability to detect change (Pearson correlations between Thrive and comparator instruments’ residualized change scores in longitudinal data, N=51).

Variable	PHQ^a^-9, total		PHQ-9, sleep item		SF^b^-20, general health item		SF-20, mental health			SF-20, physical functioning			SF-20, role functioning			SF-20, health perception		
	r	*P* value	r	*P* value	r	*P* value		r	*P* value		r	*P* value		r	*P* value		r	*P* value
Overall health	—^c^	—	—	—	.311	.03		—	—		—	—		—	—		—	—
Impact of primary condition	.404	.003	—	—	—	—		.352	.011		.091	.53		.099	.49		.276	.05
Core symptoms	.475	<.001	—	—	—	—		.485	<.001		.217	.13		.145	.31		.510	<.001
Mobility	—	—	—	—	—	—		—	—		.269	.06		—	—		—	—
Sleep	—	—	.496	<.001	—	—		—	—		—	—		—	—		—	—
Abilities	.190	.18	—	—	—	—		.125	.384		−.005	.97		.330	.02		.219	.12
Thriving	.356	.01	—	—	—	—		.389	.005		.027	.85		.058	.69		.041	.78

^a^PHQ: Patient Health Questionnaire.

^b^SF: Short-Form General Health Survey.

^c^Not applicable.

**Table 8:** Final core Thrive items.

Scale name (# of items) and item label		Item content	Response options
**Overall health (1)**			
	Overall health	Over the last month, how has your health been?	5=Excellent; 4=Very good; 3=Good; 2=Fair; 1=Poor
**Impact of primary condition (1)**			
	Condition impact	Over the last month, how much has your [primary condition] affected your life?	0=Not at all; 1=A little; 2=Some; 3=A lot
**Core symptoms (5)**			
	Pain	Please rate the severity of any pain over the past month	0=None; 1=Mild; 2=Moderate; 3=Severe
	Depressed mood	Please rate the severity of any depressed mood over the past month	0=None; 1=Mild; 2=Moderate; 3=Severe
	Anxious mood	Please rate the severity of any anxious mood over the past month	0=None; 1=Mild; 2=Moderate; 3=Severe
	Fatigue	Please rate the severity of any fatigue over the past month	0=None; 1=Mild; 2=Moderate; 3=Severe
	Stress	Please rate the severity of any stress over the past month	0=None; 1=Mild; 2=Moderate; 3=Severe
**Mobility (1)**			
	Walk	Over the last month, how well could you walk without support (such as a brace, cane, or walker)?	4=Extremely well; 3=Very well; 2=Fairly well; 1=Poorly; 0=Not at all
**Sleep (2)**			
	Fall asleep	Over the last month, how well could you fall asleep when you wanted to?	4=Extremely well; 3=Very well; 2=Fairly well; 1=Poorly; 0=Not at all
	Stay asleep	Over the last month, how well could you sleep through the night?	4=Extremely well; 3=Very well; 2=Fairly well; 1=Poorly; 0=Not at all
**Abilities (5)**			
	Think	Over the last month, how well could you think, concentrate, and remember things?	4=Extremely well; 3=Very well; 2=Fairly well; 1=Poorly; 0=Not at all
	Emotions	Over the last month, how well could you control your emotions?	4=Extremely well; 3=Very well; 2=Fairly well; 1=Poorly; 0=Not at all
	Personal needs	Over the last month, how well could you take care of your personal needs?	4=Extremely well; 3=Very well; 2=Fairly well; 1=Poorly; 0=Not at all
	Responsibilities	Over the last month, how well could you meet your responsibilities at work, school, or home?	4=Extremely well; 3=Very well; 2=Fairly well; 1=Poorly; 0=Not at all
	Social	Over the last month, how well could you participate in your favorite social and leisure activities?	4=Extremely well; 3=Very well; 2=Fairly well; 1=Poorly; 0=Not at all
**Thriving (4)**			
	Good	Over the last month, how often did you feel good about yourself?	3=All of the time; 2=Most of the time; 1=Some of the time; 0=None of the time
	Meaning	Over the last month, how often did you find meaning in your life?	3=All of the time; 2=Most of the time; 1=Some of the time; 0=None of the time
	Connect	Over the last month, how often did you feel connected to others?	3=All of the time; 2=Most of the time; 1=Some of the time; 0=None of the time
	Wanted	Over the last month, how often did you feel able to live the life you wanted?	3=All of the time; 2=Most of the time; 1=Some of the time; 0=None of the time

#### Core Symptoms

A chi-square test demonstrated that the partial credit model (PCM [39]) fit significantly better than the more parsimonious rating scale model (RSM) [40] (*P*<.001). Therefore, the PCM was utilized to evaluate rating scale functioning. First, unidimensionality, item fit, and item discriminations were evaluated. A principal component analysis (PCA) on the probability scale residuals provided support for unidimensionality (see Multimedia Appendix 2). Item fit was evaluated by examining mean square infit and outfit statistics estimated by the Rasch model. Items exhibited acceptable fit to the model [41]. Item discrimination statistics were similar, although the Pain item discriminated between persons less well than the other items (discrimination=.61; see Multimedia Appendix 2 for further details).

Andrich thresholds were ordered, providing evidence that the items’ rating scales were functioning as expected [42]. Evaluation of the person-to-item map suggested adequate coverage across the latent construct (see Multimedia Appendix 2). Next, the presence of bias was evaluated via differential item function (DIF) in WINSTEPS. DIF was considered notable if the DIF contrast estimate was >1.0 in absolute value and statistically significant [43,44]. Although the presence of DIF can suggest that an item is not fair or biased, significant DIF can also indicate that the groups truly differ on the construct being measured [44]. Results did not reveal evidence of DIF for gender or race (white and nonwhite). However, results suggested the presence of DIF for the Anxious Mood item between the autoimmune relapsing and psychiatric groups, whereby this item was easier to endorse for the autoimmune relapsing group. Internal consistency was good, and stability was excellent (Table 5). Results largely provided support for convergent validity (Table 6) and ability to detect change (Table 7).

#### Mobility

Absolute agreement of responses to the Walk item across the 3-day test-retest period was good (Table 5). This single-item scale was related as anticipated to comparator measures, providing support for convergent validity (Table 6). A positive correlation between the Walk item’s and the SF-20 Physical Functioning scale’s residualized change scores (see Table 7) provide support for the Walk item’s ability to detect change over time.

#### Sleep

The PCM did not evidence significantly better fit than the RSM, so the RSM was used to evaluate rating scale functioning. Assumptions of the model were met, and results suggested that the rating scale was performing as expected. The items did not show evidence of DIF for gender, race, or condition (autoimmune relapsing, psychiatric, or neurodegenerative). Internal consistency was acceptable, and stability was good (Table 5). Due to shared content, a Pearson correlation between the PHQ-9 and Sleep scale was calculated to evaluate convergent validity of the Sleep scale. Results provided support for convergent validity. Finally, the positive correlation between Sleep and the PHQ-9 Sleep item’s residualized change scores provides evidence of ability to detect change over time (Table 7).

#### Abilities

Of the Abilities items, 1 (“Over the last month, how well could you live the life you wanted to live?”) was removed because of conceptual redundancy with another item (“Over the last month, how often did you feel able to live life you wanted?”).

A global chi-square fit test demonstrated that the PCM fit significantly better than the RSM (*P*<.001). Therefore, the PCM was utilized to evaluate rating scale functioning. Results from a PCA on the probability scale residuals provided support for unidimensionality. The items exhibited acceptable item fit and similar item discriminations.

The items’ rating scales were functioning as expected, and examination of the person-to-item map suggests adequate coverage. The items did not evidence DIF for gender or race. However, results suggested the presence of DIF for the Think item between the neurodegenerative group and the autoimmune group, whereby this item was easier to endorse for patients with neurodegenerative conditions. Internal consistency was good, and stability was excellent (Table 5). Pearson correlations provided support for convergent validity (Table 6). Results largely provided support for ability to detect change (Table 7). However, the residualized change scores for Abilities and SF-20 Physical Functioning evidenced a near-zero correlation. Evaluation of the SF-20 Physical Functioning composite reveals that items reflect physical mobility and ability to engage in vigorous physical activity (eg, lifting heavy objects, running, walking, walking uphill, bending, etc). Therefore, it is not surprising that change in the 2 scales over time were not related.

#### Thriving

A chi-square test demonstrated that the PCM fit significantly better than the RSM (*P*<.001). Therefore, the PCM was utilized to evaluate rating scale functioning. Of the items, 1 (“Over the last month, how often did you stick to the health habits you wanted to?”) was removed because of poor model fit and discrimination (.25). Following removal of this item, another item (“Over the last month, how often did you feel able to take charge of your health?”) was also removed because of poor discrimination (.67). The remaining items evidenced acceptable levels of fit [41] and discrimination [45], as well as unidimensionality based on results from a PCA of the probability scale residuals. Results suggested that the items’ rating scales were functioning as expected.

Next, for purposes of reducing the scale length, the research team utilized theoretical (review of item content) and empirical (person-to-item map, interitem correlations) rationale to identify items for removal. As a result, 4 additional Thriving items were removed (“Over the last month, how often did you feel confident that you could handle your life?,” “Over the last month, how often did you see yourself as a worthwhile person?,” “Over the last month, how often did you feel effective?,” and “Over the last month, how often did you feel you were thriving?”). Removing these items did not result in substantial loss of reliability (from a person reliability coefficient of .92 to a person reliability coefficient of .86). The remaining 4 items evidenced good person-to-item coverage and did not evidence DIF for gender, race, or condition.

Internal consistency and stability were good (Table 5). Pearson correlations largely provided support for convergent validity (Table 6) and ability to detect change (Table 7). The PHQ-9 and SF-20 Mental Health scales’ residualized change scores were significantly related to change in Thriving scores over the 30-day period, whereas near-zero correlations were observed between change in Thriving and the remaining SF-20 scales.

### Scoring

Scores for the multi-item scales (Core Symptoms, Sleep, Abilities, and Thriving) are calculated by taking the average of the items. Whether or not scores are calculated when data are missing depends on how the instrument is being used. For example, PatientsLikeMe members can complete Thrive on a monthly basis to track their functioning, and composites for the Thrive domains can be calculated with missing data so long as 80% of items are completed for each domain. Of course, calculating a score with missing items can increase measurement error. Therefore, whenever possible, patients should be encouraged to answer as many items as they feel comfortable answering.

## Discussion

### Principal Findings

PROs have the potential to move the locus of control in health care from institutions and professionals to patients themselves by enabling digital health tools that track and predict outcomes, alert their health team, support shared decision making, enable learning from their peer group, underpin systematic self-experimentation, and let them continually participate in research [46]. Building tools that motivate users to *want* to come back and enter data requires PROs that pay as much respect to principles of user design and user experience as they do to psychometric validity [47]. This is a new challenge for a field more used to designing instruments on paper for researchers to administer in blinded clinical trials, but it is one we will have to address to help fight the law of attrition [48] and gather sufficient data to understand their disease and make better decisions as part of a learning health system that is *by the people, for the people* [49].

Following established best practice for instrument development [16], we have demonstrated that a novel set of PRO items (Thrive Core Items) can adequately describe the key domains of HRQoL in adults with chronic illness in a way that is positive and aspirational. Detailed psychometric analysis was used to refine the instrument to reduce burden and redundancy, and comparison with validated generic and condition-specific legacy PRO measures suggest an acceptable degree of agreement. Many PROs used in research and clinical practice today focus almost exclusively on *how bad a life* patients are living as a result of disease. Given that nearly all chronic health conditions are incurable and progressive, they serve only to document an individual’s descent into infirmity. Tools that encourage a positive mindset and support goal-setting to thrive despite symptoms and disability may well be important in encouraging patients to live their best life by seeking pleasure, engagement, and meaning [50].

During our interviews, patients consistently described *disease* only as a problem to be managed, *health* as the overall state of their bodies and minds, and *thriving* as living the life they wanted to live. Of the participants, 1 remarked:

Health incorporates disease but is bigger. Health is the ability to enjoy life with minimal impact from your conditions. It’s feeling good about life and who you are. Thriving is even more than health...it’s looking forward to each day with desire...and feeling that life is good.

After reviewing the items, most participants interviewed agreed that the Thrive Core items regarding meaning, connectedness to others, self-esteem, and coping were best at reflecting what thriving meant to them.

### Advantages of Thrive for Digital Health

Thrive contains a number of features designed to make it appealing for use in digital health. Using consistent items across multiple conditions is supportive of patients with multiple comorbidities. For example, a patient living with both PD and MDD only needs to complete information about shared domains (such as ability to sleep) once. By contrast, in our previous PRO model, a patient would have been asked to complete not only a Parkinson-specific measure (the PDRS) but also a mood-specific measure (the mood map) and a generic HR-QoL measure (PLM-QoL), with a number of additional symptoms. The burden of this battery of instruments (100 items with 3 different recall periods, 5 different response scales, and some 3252 words to read) is dramatically reduced by Thrive (19 core items plus 22 condition-specific questions [41 total] in 924 words across consistent response scales and recall periods). Question stems and response options are short and consistent, being optimal for use on mobile displays. When deployed on PatientsLikeMe, users have the option to respond “stop asking me this” for each item, which may be particularly useful for members with quadriplegia whose condition will not improve, those who feel emotionally *triggered* by certain questions, or who are in good physical health but only want to track mood or other psychological symptoms. Although fewer than 1% of real-world users choose to switch off an item (internal data), interviewees felt this option offered a greater sense of control over their own experience rather than attempting to skip an item or enter false data to skip to the next screen.

### Limitations

This study was subject to a number of limitations. Although the overall number of participants recruited was relatively large, it was a convenience sample from users of an online health community, had only a 9.5% completion rate from those invited, and there was a bias for participants to be more likely to be female and well-educated. There was significant attrition in both rounds of the 3-day retest and 30-day follow-up, which limited our ability to detect minimally important differences and may limit generalizability. Our sample was limited to English-speaking participants residing in the United States with a handful of chronic health conditions. All this limits generalizability to other populations and should be tested further. A larger, prospective, longitudinal study over a longer time course would have been preferable to establish minimally important differences and sensitivity to change. Although Thrive will be deployed with multiple items relating to both the *Impact of Primary Condition* and additional *Impact of additional conditions* related to their comorbidities, this study only asked about a single condition. This may have obscured the impact of important comorbidities.

The number of cognitive interviews conducted was a *total* of 12 participants; arguably we might have interviewed 12 patients for *each* of the 9 condition groups represented in the sample [51]. However, as we were developing a measure for chronic illness more generally rather than specific conditions, this was considered adequate, and both of our interviewers felt we had achieved *saturation* [52]. Interviewing over 100 participants was also considered infeasible in the time and budget allowed.

All participant data were self-reported rather than being independently validated, though previous studies suggest a high degree of agreement between patient self-report of diagnosis and confirmation via, for example, insurance claims [53]. Some of the condition-specific comparator measures used on PatientsLikeMe, and by extension, this study, were unvalidated—they were tested to further our plans to remove them from our online community but do not provide as robust tests as a validated measure would have achieved. However, the use of the widely used SF-20 and PHQ-9 make up for this shortcoming to some degree. Owing to the number of conditions and comparator measures, our reporting of convergent validity was necessarily more simplistic than desirable. Small samples for condition-specific measures such as the PLM-QoL, MSRS, and ALSFRS-R relative to the PHQ-9 or SF-20 may have obscured the strength of relationships for comparative validity in the former. Next steps for validation include deployment of the Core Thrive Items in a more representative sample of US citizens and testing of disease-specific versions of Thrive in clinical settings alongside ClinROs and objective measures such as blood tests.

### Modularity for Expansion and Future Research

Analysis of comparative validity suggests that although there are moderate-strong correlations with overlapping domains from other instruments, it is unlikely that the Core items represent complete *coverage* of all the important domains for every condition. For example, there were only moderate correlations between the Mobility and Abilities scales with the ALSFRS-R [12], and clinical experience tells us that a measure that fails to take speech, swallowing, feeding, or breathing ability into account would be missing key data for understanding patients and their disease.

**Figure 2:**
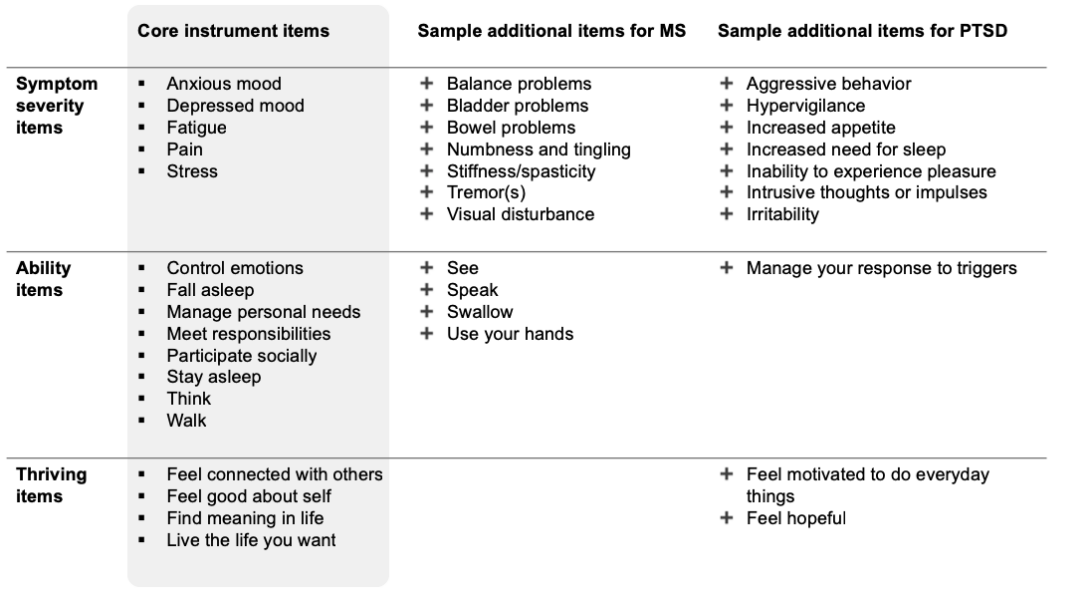
Sample additional items for 2 conditions based on health care professional review. MS: multiple sclerosis; PTSD: posttraumatic stress disorder.

Work is already in progress to describe the development of condition-specific item banks that can be interspersed with the Thrive Core Items (Figure 2). Review of existing PROs, the clinical literature, and the patient-added symptoms of existing PatientsLikeMe users have been used to add additional domains such as *tremor* as a symptom in Parkinson disease, or *breathing* as an ability in ALS, for instance. Future studies will describe condition-specific validation of Thrive-Condition Instruments such as *Thrive-ALS* against legacy measures such as the ALSFRS-R [12] in more detail, with the addition of clinical and other objective biomarkers where possible. Such clinical work will also be useful in establishing minimally important differences for changes on different Thrive subscores over time and in response to treatment. The Thrive Core Items are available to members of PatientsLikeMe.com as a *MonthlyMe* interview, and although the psychometric validation described herein is probably sufficient to support patient self-tracking and visualization of individual items and subscores (eg, showing how an individual compares to a group of patients like them, or showing relationships between different variables), further condition-specific work is needed to confirm the tool’s validity for clinical management or proving a treatment effect in clinical trial.

Work with partners may also involve translation into other languages (such as Mandarin Chinese) and deployment through mobile messaging platforms (such as WeChat) as part of wellness apps. Finally, future work will consider the role of treatment side effects and treatment burden as key aspects of thriving despite illness [54]. We offer the use of Thrive under Creative Commons Attribution ShareAlike International (CCSA-4.0) so that others can deploy and adapt it in their work to measure what matters most to people. Although there is always the risk in taking this approach that some users may use the instrument inappropriately (eg, by adding poor quality items or mistranslating into other languages), we believe the tangible benefits of making an instrument freely available outweigh the theoretical harms.

### Conclusions

Validation is a continuous and iterative process. This study describing the development and testing of the Thrive Core Set items is the first step on a path that includes replacing all the PROs on PatientsLikeMe, testing against putative biomarkers of disease progression, and deployment on third party digital health platforms. We hope Thrive will be a key resource in the digitization of human health to improve longevity and well-being for all.

## Appendix

Multimedia Appendix 1 Consolidated Standards of Reporting Trials flow diagrams.

Multimedia Appendix 2 Detailed round 1 psychometric analysis.

## Abbreviations

ALS: amyotrophic lateral sclerosis
ALSFRS-R: ALS functional rating scale revised
ClinRO: clinician-reported outcome
DIF: differential item function
GAD: generalized anxiety disorder
HRQoL: health-related quality of life
MDD: major depressive disorder
MS: multiple sclerosis
MSRS: multiple sclerosis rating scale
PCA: principal component analysis
PCM: partial credit model
PD: Parkinson disease
PDRS: Parkinson’s disease rating scale
PHQ: Patient Health Questionnaire
PRO: patient-reported outcome
PTSD: posttraumatic stress disorder
RSM: rating scale model
SF: Short-Form General Health Survey
SLE: systemic lupus erythematosus
