# Supplementary material for: A Modular Health-Related Quality of Life Instrument for Electronic Assessment and Treatment Monitoring: Web-Based Development and Psychometric Validation of Core Thrive Items
Source: J Med Internet Res. 2019 Jan 25;21(1):e12075. doi: 10.2196/12075 (PMC6367664; doi:10.2196/12075)

SUPPLEMENTARY APPENDIX 1

CONSORT Flow Diagrams

Round 1: Test

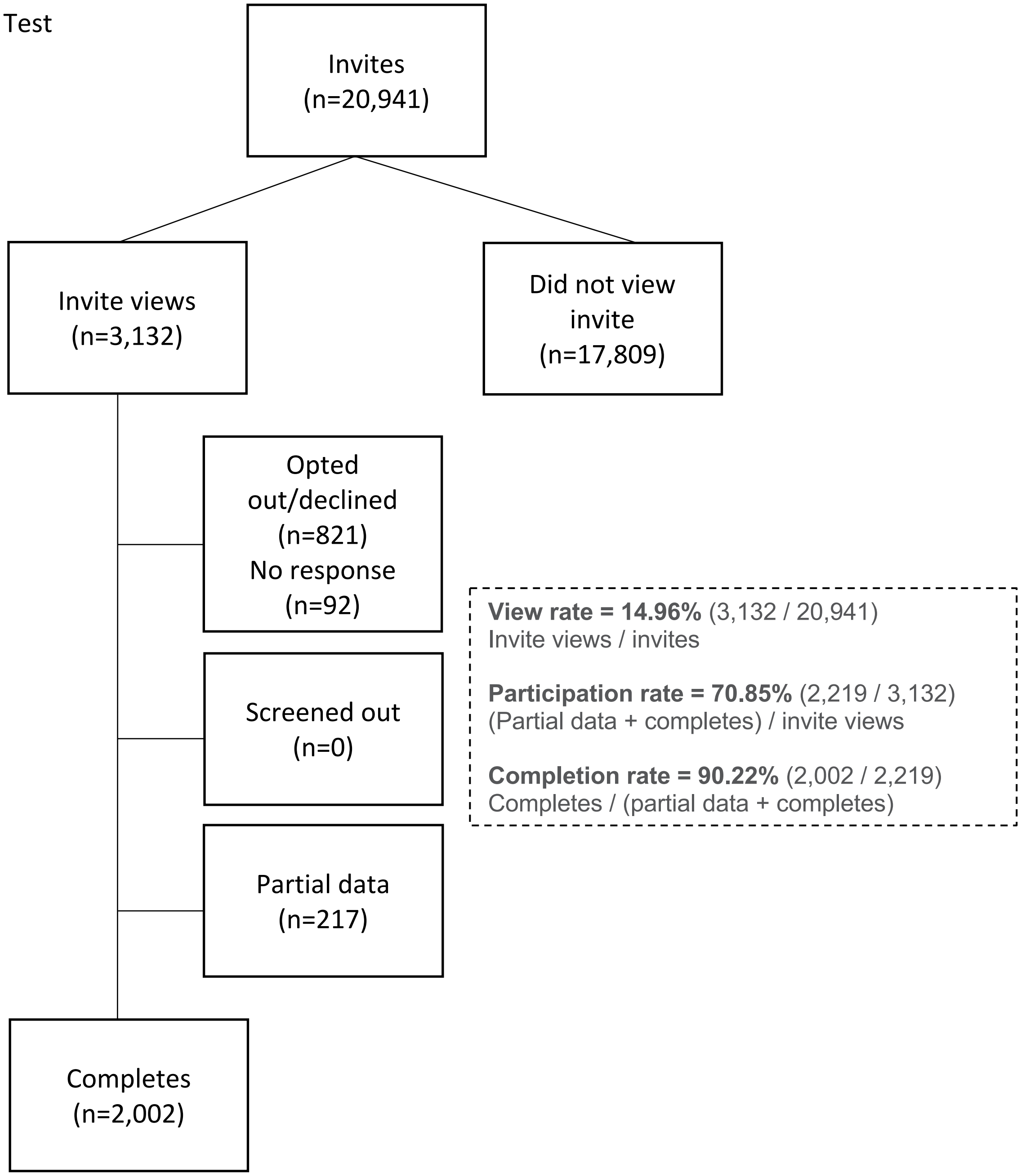

Round 1: Re-Test

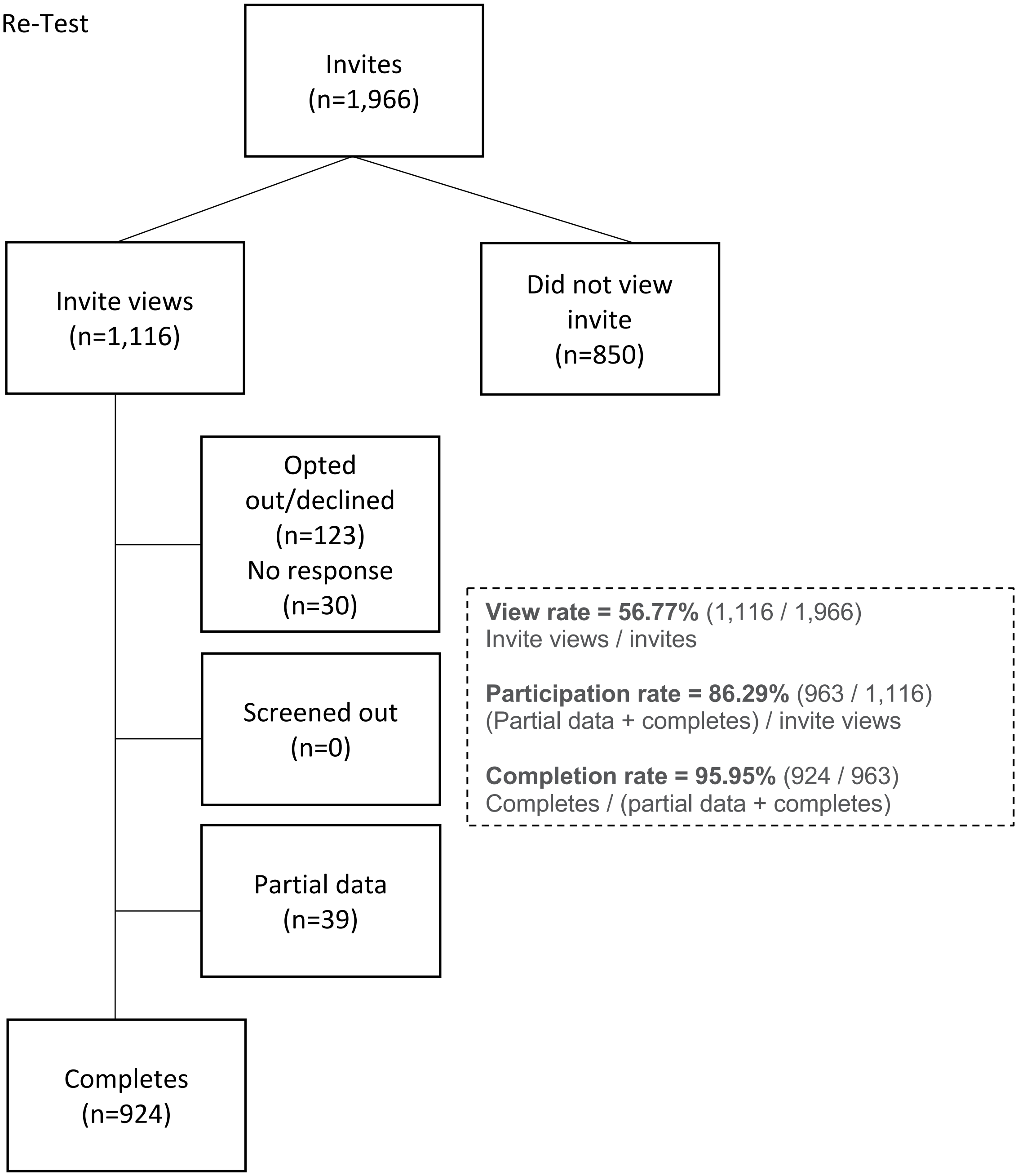

Round 1: 30-day Re-Test

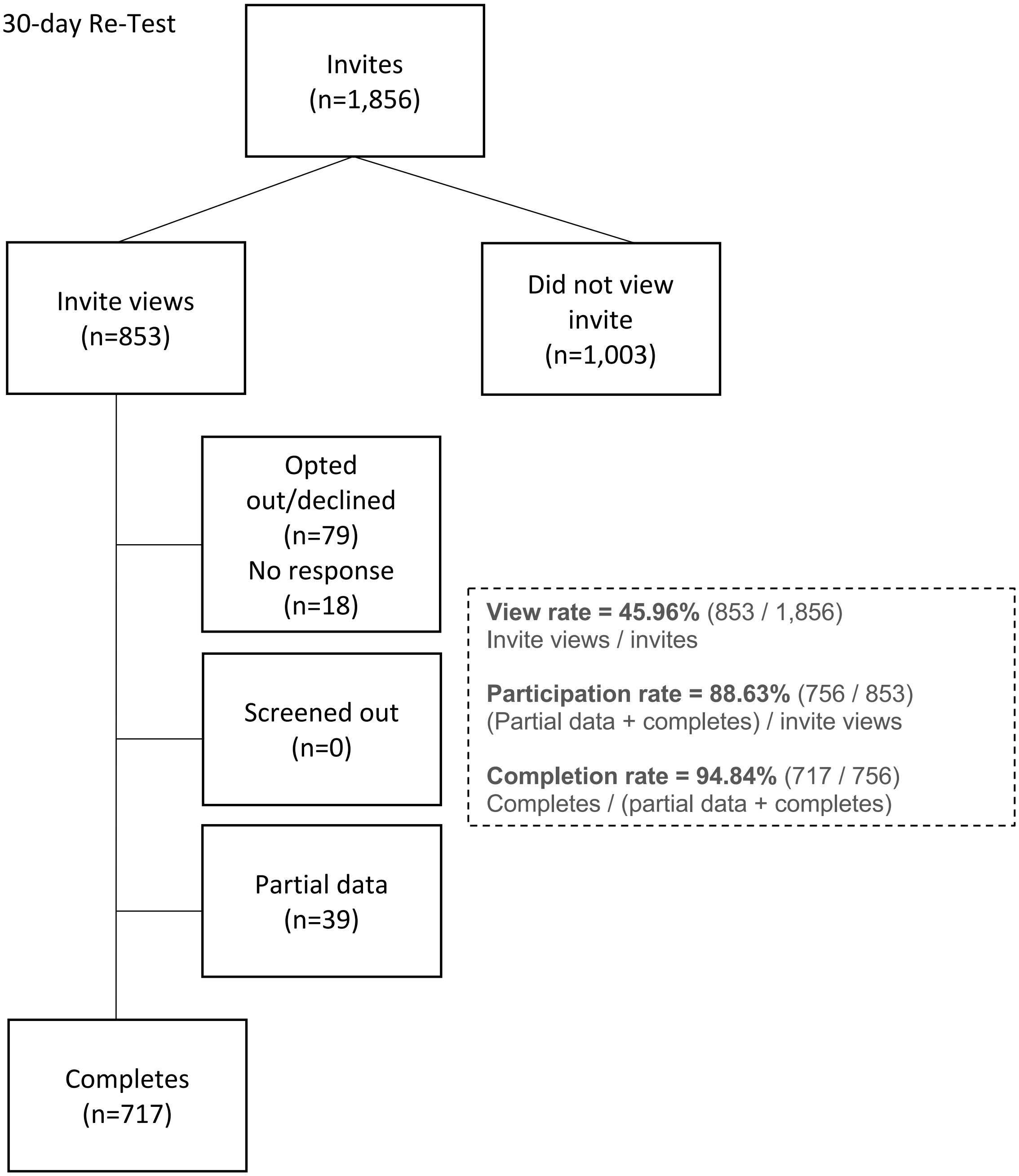

Round 2: Test

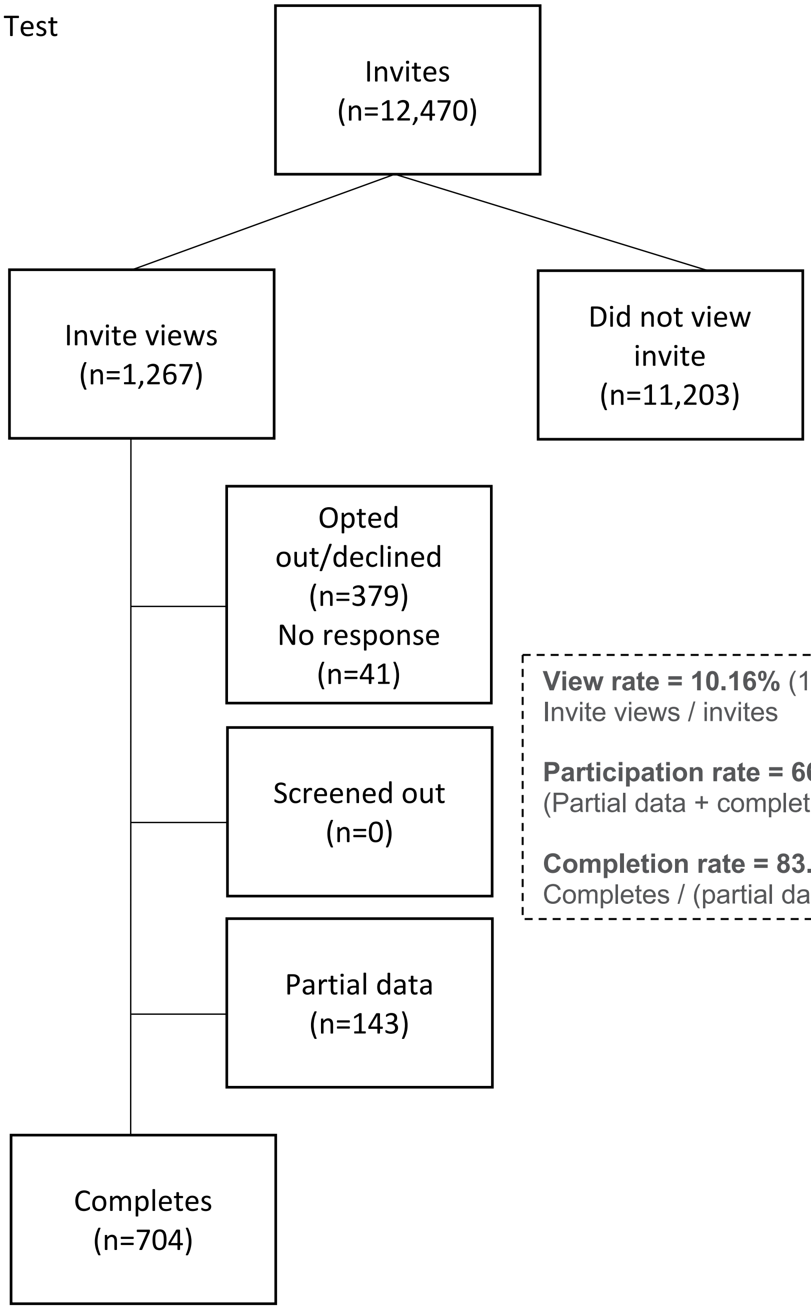

View rate = 10.16% (1,267 / 12,470)
Invite views / invites

Participation rate = 66.85% (847 / 1,267)
(Partial data + completes) / invite views

Completion rate = 83.12% (704 / 847)
Completes / (partial data + completes)

Round 2: Re-Test

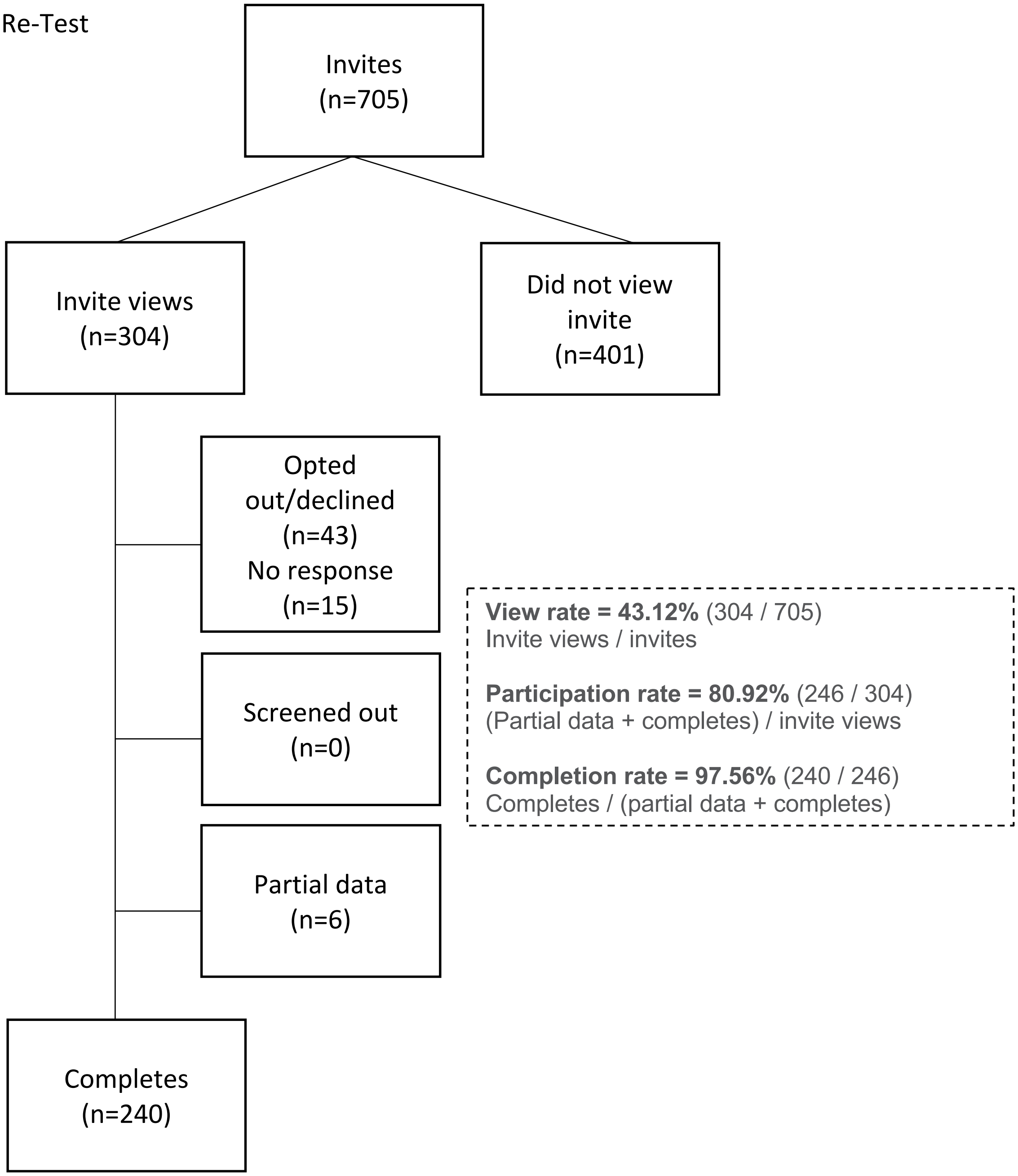

Round 2: 30 day Re-Test

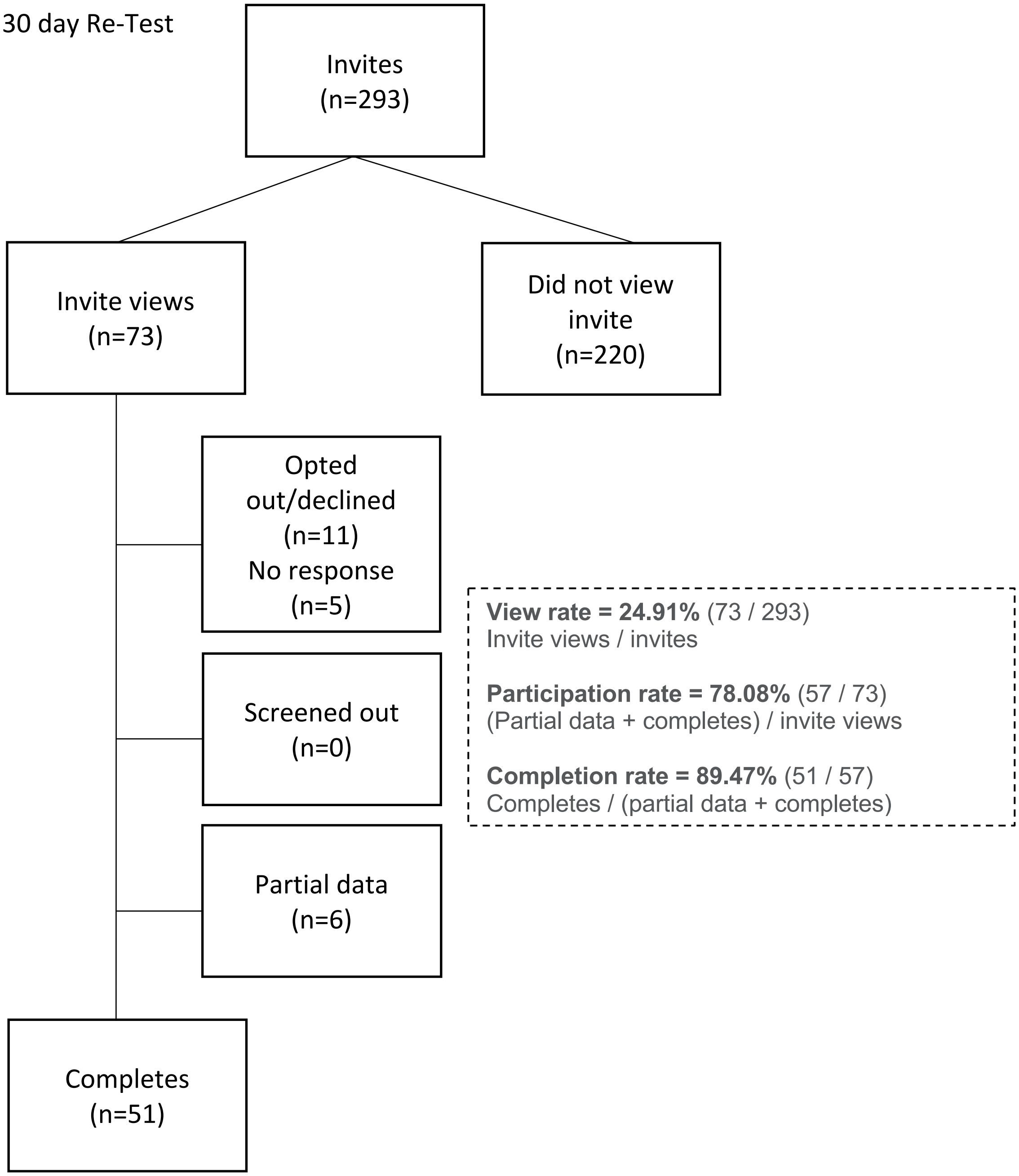

Supplement: Multimedia Appendix 1 [file jmir_v21i1e12075_app1.pdf]
