# Supplementary material for: A Modular Health-Related Quality of Life Instrument for Electronic Assessment and Treatment Monitoring: Web-Based Development and Psychometric Validation of Core Thrive Items
Source: J Med Internet Res. 2019 Jan 25;21(1):e12075. doi: 10.2196/12075 (PMC6367664; doi:10.2196/12075)
Supplement: Multimedia Appendix 2 [file jmir_v21i1e12075_app2.pdf]

SUPPLEMENTARY APPENDIX 2

Additional Psychometric Analysis Details

ROUND 1

Summary of Results from Round 1 Empirical Evaluation

Change in Health. An intraclass correlation coefficient (ICC) was computed to estimate absolute agreement of participants' ratings of Change in Health over the 3-day test-retest period. Stability of this single-item scale was questionable (see table below).

Impact of Primary Condition. Stability of this single-item scale was good. A moderate correlation between Impact of Primary Condition and the SF-20 Health Perception scale provide support for convergent validity. Results suggested that this scale is able to detect change over time, evaluated by correlating Impact of Primary Condition and SF-20 Health Perception residualized change scores.

Severity of Treatment Side Effects. Stability of this single-item scale, calculated only with patients who reported that they were currently taking medication, was questionable.

Core Symptoms. A partial credit model (PCM; Masters, 1982) was employed in WINSTEPS to evaluate rating scale functioning. Results revealed adequate rating scale functioning, and provided evidence of unidimensionality and adequate person-to-item coverage. Differential item functioning (DIF) was found for the Pain item, whereby this item was easier to endorse for patients with primary autoimmune relapsing conditions than for patients with primary psychiatric conditions. Internal consistency was good and stability was excellent. Pearson correlations with PHQ-9, SF-20 scales, and submission number provided support for convergent validity. Correlations between the Core Symptoms scale and the PHQ-9 and SF-20 scales' residualized change scores provided support for ability to detect change.

Mobility. This single-item (ability to walk without support) scale evidenced good to excellent stability, as well as a moderate to strong correlation with the SF-20 Physical Functioning scale, providing support for convergent validity. A positive correlation between the Mobility item and the SF-20 Physical Functioning scale's residualized change scores provided evidence of ability to detect change over time.

Abilities. A PCM was employed and results revealed adequate rating scale functioning, provided support for unidimensionality, and suggested adequate person-to-item coverage. One item pertaining to mobility evidenced poor fit to the model as well as poor discrimination (-.18), suggesting that this item was degrading the measurement model and was removed. The remaining 8 items did not evidence significant DIF for gender, race, or condition (i.e., psychiatric, neurodegenerative, and autoimmune relapsing). Internal consistency was good and stability was excellent. Results provided support for convergent validity and ability to detect change over time.

Thriving. A PCM was employed and results revealed adequate rating scale functioning, provided support for unidimensionality, and suggested adequate person-to-item coverage. Items did not evidence significant DIF for gender, race, or condition. Internal consistency was excellent and stability was good. Results provided support for convergent validity and ability to detect change over time.

Summary of Results from Round 1

			Convergent Validity (Pearson correlation (r, p))					
Scale (N of items)	Internal Consistency Reliability (α¿	Test-Retest Reliability (absolute ICC, p)	PHQ-9 Total	SF-20 Mental Health	SF-20 Physical Functioning	SF-20 Role Functioning	SF-20 Health Perception
Change in Health (1)	--	.611 (<.001)	--	--	--	--	--
Impact of Primary Condition (1)	--	.759 (<.001)	--	--	--	--	-.503 (<.001)
Severity of Treatment Side Effects (1)	--	.637 (<.001)	--	--	--	--	--
Core Symptoms (5)	.833	.911 (<.001)	.776 (<.001)	-.787 (<.001)	-.254 (<.001)	-.300 (<.001)	-.598 (<.001)
Mobility (1)	--	.878 (<.001)	--	--	.635 (<.001)	--	--
Abilities (8)	.882	.908 (<.001)	-.746 (<.001)	.694 (<.001)	.464 (<.001)	.483 (<.001)	.715 (<.001)
Thriving (6)	.924	.891 (<.001)	-.791 (<.001)	.829 (<.001)	.209 (<.001)	.280 (<.001)	.557 (<.001)

ROUND 2

Results from Round 2: Evaluation of Dimensionality

Core Symptoms. In Rasch modeling, dimensionality is evaluated by employing a principal components analysis (PCA) on the probability scale residuals. After extracting the primary Rasch dimension, results did not suggest the presence of multiple dimensions (unexplained variance in the first contrast =1.7, with a score <2.0 suggesting absence of a meaningful second dimension per Linacre, J. M. (2018). Dimensionality: Contrasts & variances. https://www.winsteps.com/winman/principalcomponents.htm). The Rasch model explained 56.3% of the raw variance, providing additional support for unidimensionality.

Abilities. A PCA was conducted on the probability scale residuals in WINSTEPS. The unexplained variance in the first contrast was 1.9, and the Rasch model explained 64.4% of the raw variance. These findings support unidimensionality.

Thriving. A PCA was conducted on the probability scale residuals in WINSTEPS. Results revealed that the Rasch model explained 71.3% of the raw variance. Further, the unexplained variance in the first contrast was 1.4, providing support for unidimensionality.

Round 2: Item Difficulty, Fit Statistics, and Response Category Thresholds

Thrive Scale	Item Label	Difficulty	Infit MNSQ	Outfit MNSQ	T_1	T_2	T_3	T_4
Core Symptoms	Pain	.29	1.33	1.33	-1.87	-.34	2.21	--
	Depressed Mood	.66	.84	.83	-2.05	-.15	2.20	--
	Anxious Mood	.56	.85	.86	-2.05	-.11	2.16	--
	Fatigue	-.97	1.08	1.12	-2.44	-.02	2.46	--
	Stress	-.54	.88	.86	-2.81	.34	2.47	--
Sleep[1]	Fall Asleep	-.53	.97	.96	-5.38	-1.33	1.95	4.76
	Stay Asleep	.53	1.01	1.00	-5.38	-1.33	1.95	4.76
Abilities	Think	-.13	1.16	1.16	-5.06	-.64	1.86	3.83
	Emotions	-.09	1.14	1.14	-3.90	-1.28	1.57	3.61
	Personal Needs	-1.26	.92	.90	-4.31	-.72	1.68	3.35
	Responsibilities	.26	.81	.81	-3.20	-1.08	1.28	3.00
	Social	1.22	.94	.95	-3.22	-1.03	1.10	3.14
Thriving	Good	-.33	.96	.92	-5.09	.33	4.76	--
	Meaning	-.97	.83	.79	-4.49	.41	4.08	--
	Connect	-.56	1.05	1.02	-5.27	.61	4.66	--

	Wanted	1.85	1.12	1.20	-4.51	-.03	4.54	--

MNSQ = mean-square
T=threshold
Data were generated using WINSTEPS software
[1] Note that unlike the other Thrive scales, the PCM did not fit significantly better than the RSM and therefore the more parsimonious RSM was utilized for the Sleep scale (hence the thresholds are identical across the two Sleep items)

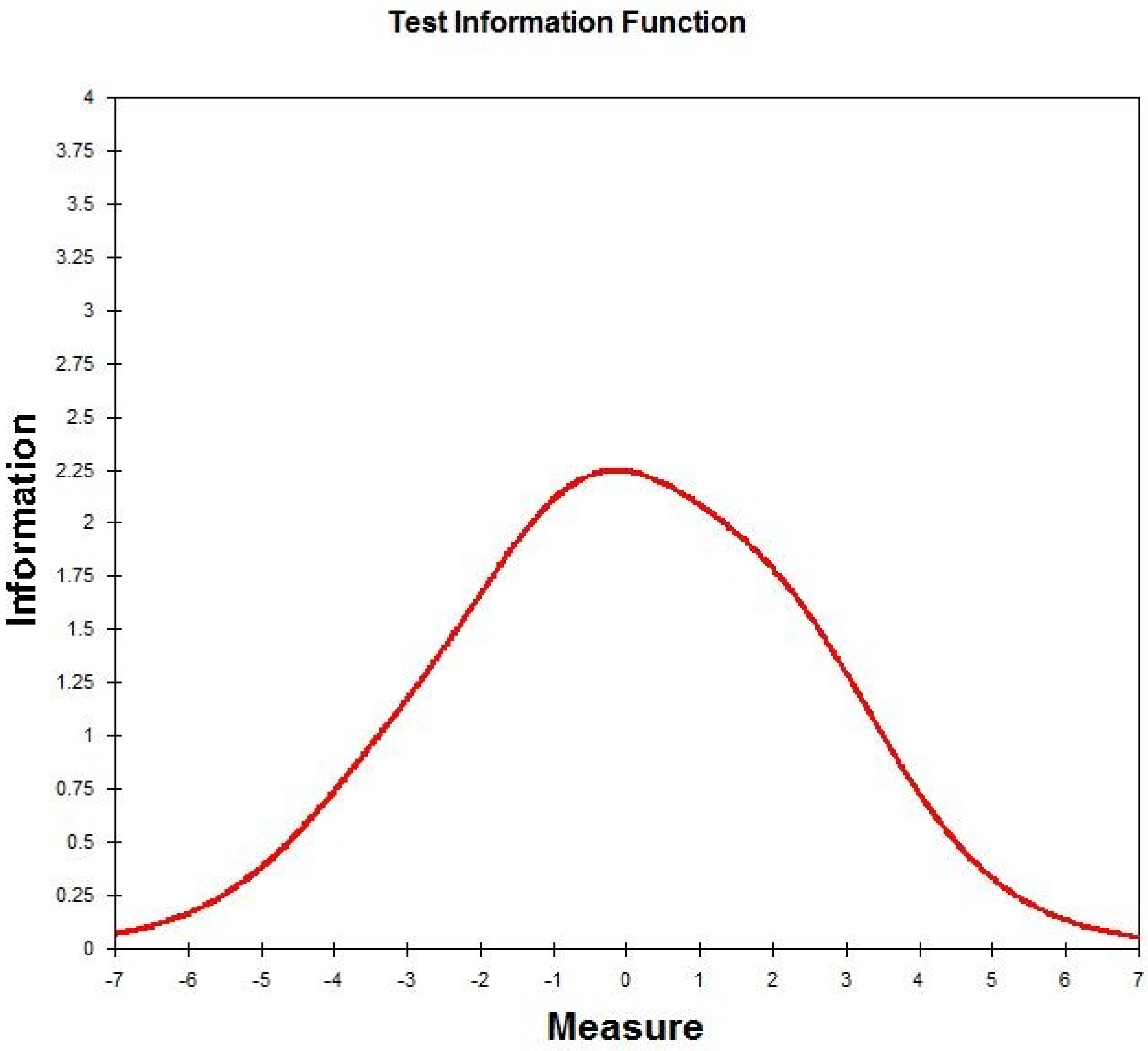

Test Information Function for the Core Symptoms scale (variance of latent variable = 3.766)

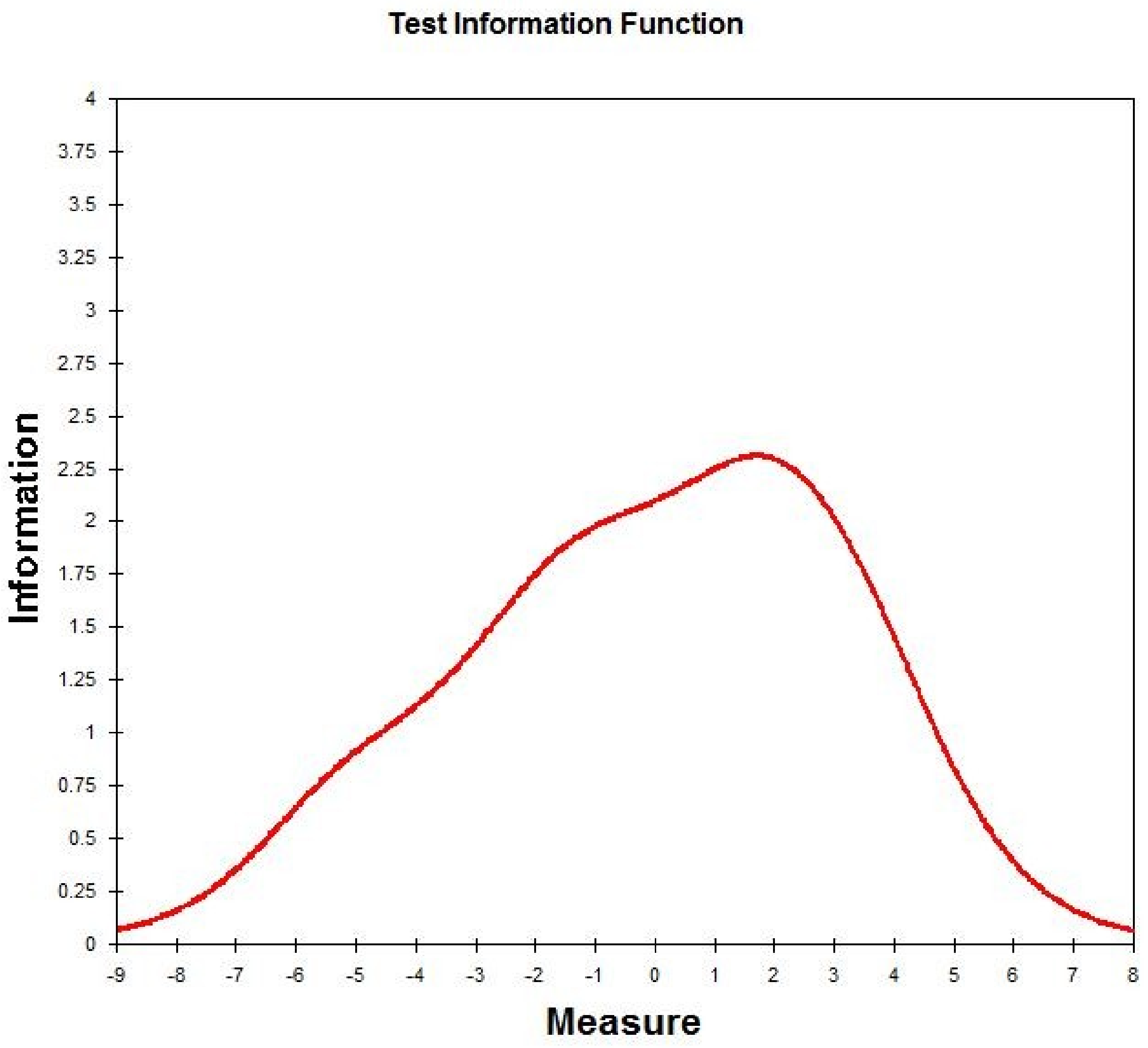

Test Information Function for the Abilities Scale (variance of latent variable =4.378)

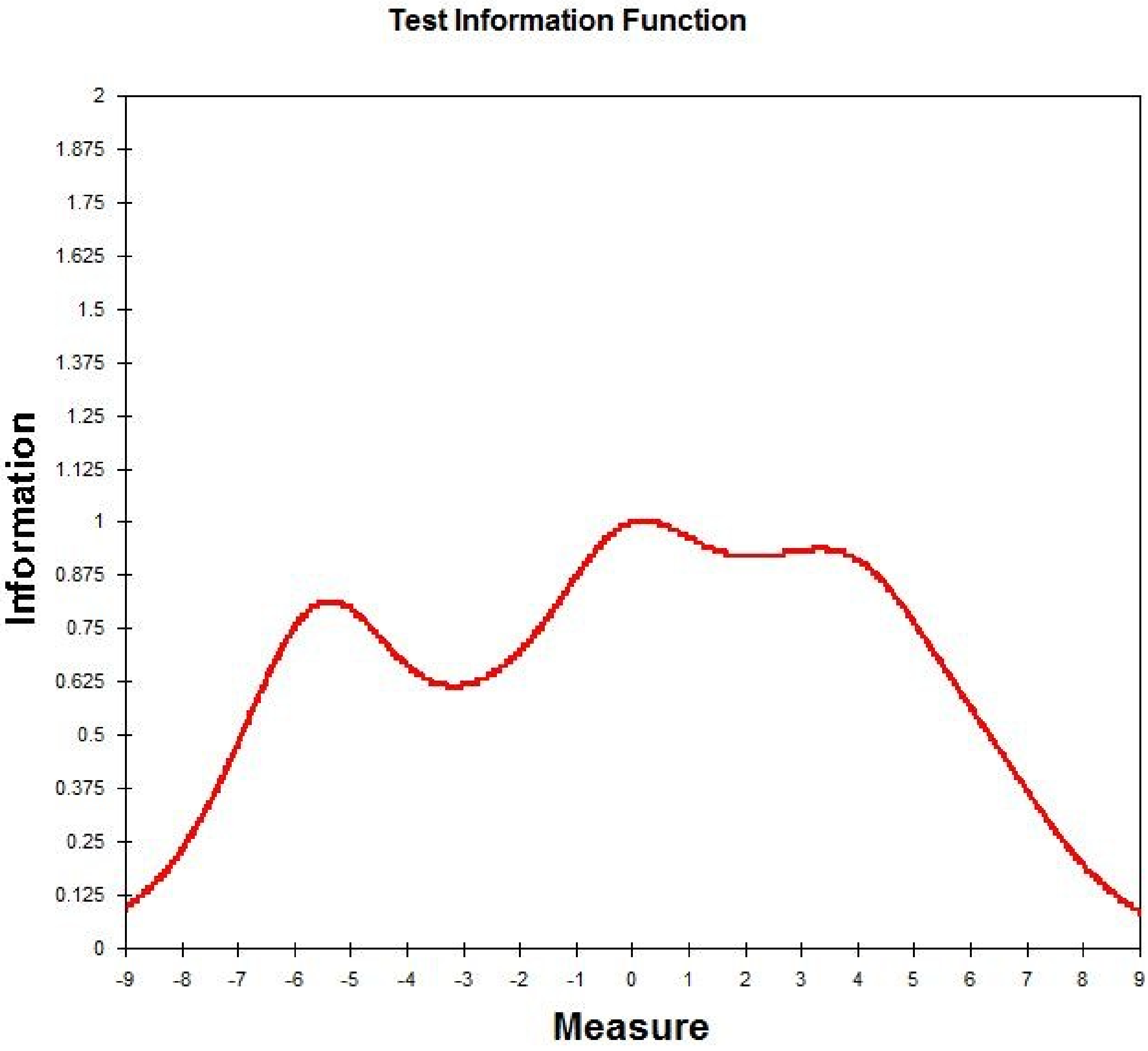

Test Information Function for the Thriving scale (variance of latent variable =12.970)

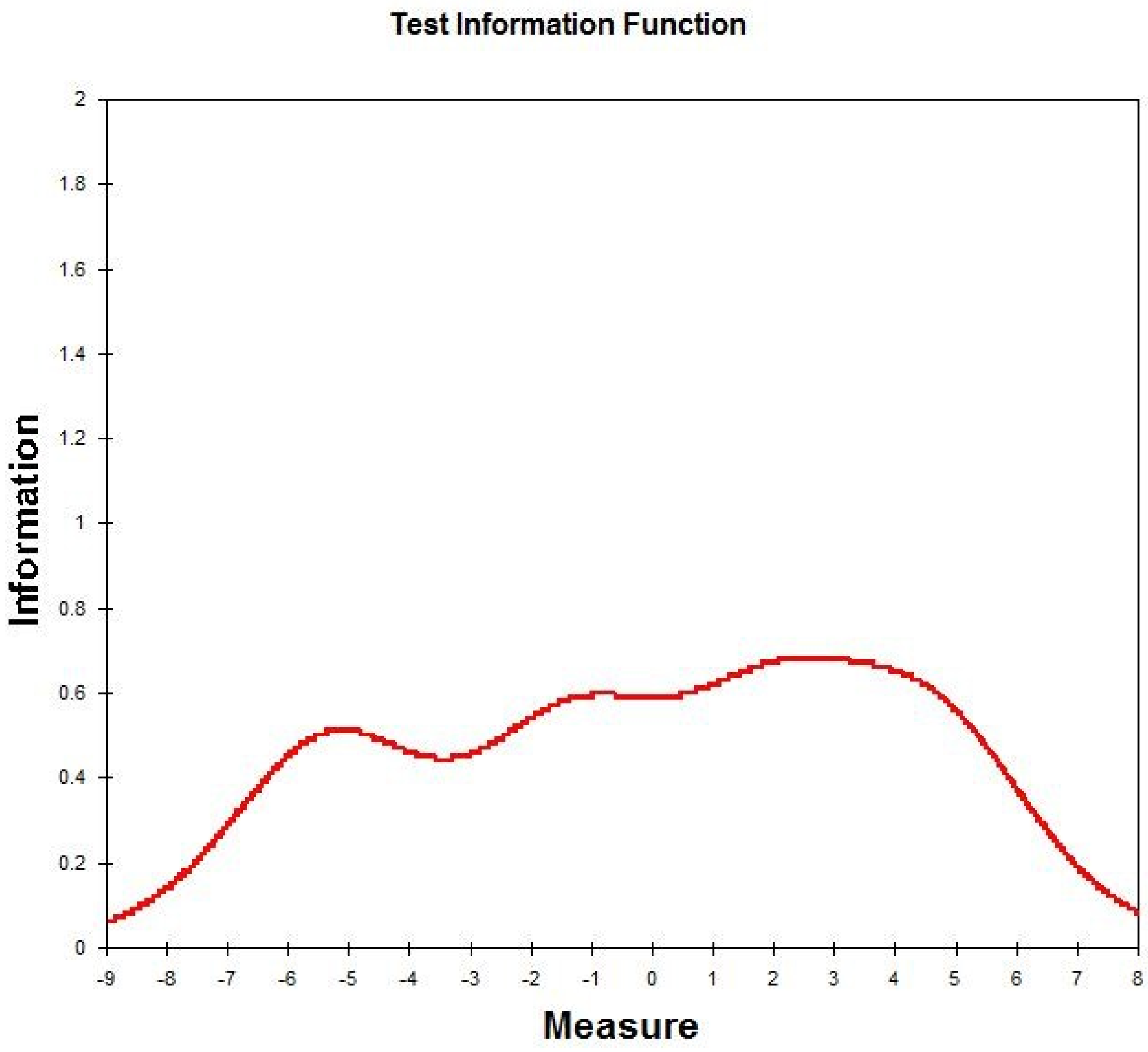

Test Information Function for the Sleep scale (variance of latent variable = 10.969)

```
MEASURE    PERSON - MAP - ITEM - 50% Cumulative probabilities (Rasch-Thurstone thresholds)
                <more>|
    4             .###  +
                 #####  |
                       T|
                        |
                        |
                        |
                        |
    3                   +                          DEP_MOOD.3
            .#######    |                          ANX_MOOD.3
                        |
                        |                          PAIN    .3
                        |
            .#######    |
                        |
    2                  S+                          STRESS  .3
                        |
          ##########    |
                        |                          FATIGUE .3
                        |
                        |T
       .###########     |
    1                   +
                        |
       .###########     |S
                        |             DEP_MOOD.2
                       M|             ANX_MOOD.2
       .###########     |
                        |
    0                   +M            PAIN    .2
      .############     |
                        |             STRESS  .2
                        |
                        |
           .########    |S
                        |
   -1                   +             FATIGUE .2
          .#########    |
                       S|T
                        |
             .######    |  ANX_MOOD.1
                           DEP_MOOD.1
                        |  PAIN    .1
                        |
   -2                   +
               .####    |
                        |
                        |
                        |
                        |
                        |
   -3            ##### T+
                        |
                        |
                        |  FATIGUE .1
                           STRESS  .1
                        |
                        |
                        |
   -4               ##  +
                <less>|
 EACH "#" IS 6. EACH "." IS 1 TO 5
```

Person to Item Map for the Core Symptoms scale

```
MEASURE    PERSON - MAP - ITEM - 50% Cumulative probabilities (Rasch-Thurstone thresholds)
               <more>|
    6            .#  +
                     |
                     |
                     |
                     |
    5           .##  +
                     |
                     |
                     |                                      SOCIAL  .4
                .## T|
    4                +
                     |                                      THINK   .4
                     |                                      EMOTIONS.4
              .####  |                                      RESP    .4
                     |
    3           .##  +
                     |
                     |
             .#####  |                          SOCIAL  .3
                    S|                                      NEEDS   .4
    2       #######  +
                     |
          #########  |T                         THINK   .3
                     |                          EMOTIONS.3
                                                RESP    .3
            #######  |
    1                +
        .##########  |S
                     |
                     |                          NEEDS   .3
        ########### M|              SOCIAL  .2
    0                +M
          .########  |
                     |
       .###########  |
                     |S             RESP    .2
                                    THINK   .2
   -1                +
       ############  |
                     |              EMOTIONS.2
                     |T
            .###### S|
   -2                +  SOCIAL  .1  NEEDS   .2
                     |
           .#######  |
                     |
                     |
   -3         #####  +  RESP    .1
                     |
                     |
                    T|
                .##  |
   -4                +  EMOTIONS.1
                     |
                     |
                  .  |
                     |
   -5                +
                     |  THINK   .1
                     |
                     |  NEEDS   .1
                     |
   -6             .  +
               <less>|
 EACH "#" IS 6. EACH "." IS 1 TO 5
```

Person to Item Map for the Abilities scale

```
MEASURE    PERSON - MAP - ITEM - 50% Cumulative probabilities (Rasch-Thurstone thresholds)
               <more>|
    7            .#  +
                     |
                     |                        WANTED .3
                     |
    6            ##  +
                     |
                    T|
                     |
    5                +
                     |
                ###  |                        GOOD   .3
                     |
    4                +                        CONNECT.3
                     |
             .#####  |
                     |                        MEANING.3
    3                +
                     |
                     |
          ######### S|T
    2                +
                     |             WANTED .2
                     |
           .#######  |
    1                +S
                     |
                     |
          .########  |
    0                +M            CONNECT.2
                                   GOOD   .2
                     |
                     |             MEANING.2
          #########  |
   -1               M+S
                     |
                     |
                     |
   -2 .############  +
                     |T
                     |
                     |  WANTED .1
   -3                +
                     |
        ###########  |
                     |
   -4               S+
                     |
                     |
                     |
   -5       .######  +
                     |
                     |  GOOD   .1
                        MEANING.1
                     |  CONNECT.1
   -6                +
            #######  |
                     |
                     |
   -7         .####  +
               <less>|
 EACH "#" IS 8. EACH "." IS 1 TO 7
```

Person to Item Map for the Thriving scale

```
MEASURE    PERSON - MAP - ITEM - 50% Cumulative probabilities (Rasch-Thurstone thresholds)
                <more>|
    6             .#  +
                      |
                      |
                      |                                      STAY_SLE.4
                      |
    5             ##  +
                     T|
                      |
                      |
                      |                                      FALL_SLE.4
    4                 +
                      |
                      |
              .#####  |
                      |
    3                 +
                      |
                      |
                      |                          STAY_SLE.3
                      |
    2                S+
             .######  |
                      |
                      |                          FALL_SLE.3
                      |
    1                 +T
                      |
                      |S
                      |
          .#########  |
    0                 +M
                      |
                      |
                      |S
                     M|             STAY_SLE.2
   -1                 +T
       .############  |
                      |
                      |             FALL_SLE.2
   -2                 +
                      |
                      |
                      |
                      |
   -3                 +
                      |
        .###########  |
                      |
                     S|
   -4                 +
                      |
                      |
                      |
                      |  STAY_SLE.1
   -5                 +
                      |
             .######  |
                      |
                      |
   -6            .##  +  FALL_SLE.1
                <less>|
 EACH "#" IS 12. EACH "." IS 1 TO 11
```

Person to Item Map for the Sleep scale

Summary of Item Status Based on Round 2 Empirical Evaluation

Thrive Scale	Item Label	Status
Overall Health	Overall Health	Retained
Change in Health	Change in Health	Discarded
Impact of Primary Condition	Impact of Primary Condition	Retained
Core Symptoms	Pain	Retained
	Depressed Mood	Retained
	Anxious Mood	Retained
	Fatigue	Retained
	Stress	Retained
Mobility	Walk	Retained
Sleep	Fall Asleep	Retained
	Stay Asleep	Retained
Abilities	Think	Retained
	Emotions	Retained
	Personal Needs	Retained
	Responsibilities	Retained
	Social	Retained
	Live Life	Discarded
Thriving	Habits	Discarded
	Life	Discarded
	Worth	Discarded
	Good	Retained
	Effect	Discarded
	Meaning	Retained
	Connect	Retained
	Able	Discarded
	Wanted	Retained
	Thrive	Discarded
